# A conserved motif promotes HpaB‐regulated export of type III effectors from *Xanthomonas*

**DOI:** 10.1111/mpp.12725

**Published:** 2018-10-16

**Authors:** Heike Prochaska, Sabine Thieme, Sebastian Daum, Jan Grau, Cornelius Schmidtke, Magnus Hallensleben, Peter John, Kirsten Bacia, Ulla Bonas

**Affiliations:** ^1^ Institute for Biology, Department of Genetics Martin Luther University Halle‐Wittenberg Halle (Saale) 06120 Germany; ^2^ Institute for Chemistry, Department of Biophysical Chemistry Martin Luther University Halle‐Wittenberg Halle (Saale) 06120 Germany; ^3^ Institute for Informatics, Department of Bioinformatics Martin Luther University Halle‐Wittenberg Halle (Saale) 06120 Germany

**Keywords:** AvrBs1, AvrBsT, cardiolipin, liposomes, type III secretion

## Abstract

The type III secretion (T3S) system, an essential pathogenicity factor in most Gram‐negative plant‐pathogenic bacteria, injects bacterial effector proteins directly into the plant cell cytosol. Here, the type III effectors (T3Es) manipulate host cell processes to suppress defence and establish appropriate conditions for bacterial multiplication in the intercellular spaces of the plant tissue. T3E export depends on a secretion signal which is also present in ‘non‐effectors’. The latter are secreted extracellular components of the T3S apparatus, but are not translocated into the plant cell. How the T3S system discriminates between T3Es and non‐effectors is still enigmatic. Previously, we have identified a putative translocation motif (TrM) in several T3Es from *Xanthomonas campestris* pv. *vesicatoria *(*Xcv*). Here, we analysed the TrM of the *Xcv* effector XopB in detail. Mutation studies showed that the proline/arginine‐rich motif is required for efficient type III‐dependent secretion and translocation of XopB and determines the dependence of XopB transport on the general T3S chaperone HpaB. Similar results were obtained for other effectors from *Xcv*. As the arginine residues of the TrM mediate specific binding of XopB to cardiolipin, one of the major lipid components in *Xanthomonas* membranes, we assume that the association of T3Es to the bacterial membrane prior to secretion supports type III‐dependent export.

## INTRODUCTION

Most Gram‐negative bacterial phytopathogens possess a type III secretion (T3S) system as a key pathogenicity factor that translocates type III effector (T3E) proteins directly into the plant cell cytosol. Here, T3Es manipulate cell processes to the benefit of the pathogen, e.g. by suppression of plant immune reactions. In resistant plants, individual effectors can be recognized by corresponding resistance (*R*) genes, triggering defence reactions which often culminate in the hypersensitive response (HR), a fast local cell death reaction that inhibits further pathogen spread (Büttner, 2016; Mur *et al*., 2008).

We have studied T3Es and T3S in *Xanthomonas campestris* pv. *vesicatoria* (*Xcv*), the causal agent of bacterial spot disease on pepper and tomato plants. *Xcv* enters the plant tissue via natural openings or wounds and multiplies extracellularly in the intercellular spaces. T3Es are designated as avirulence (Avr) proteins if they were identified by their recognition in resistant plants, or as Xops (*Xanthomonas* outer proteins). Examples include ‘transcription activator‐like effectors’ (TALEs; Boch and Bonas, 2010), e.g. AvrBs3, the type member of the family, which localizes to the plant cell nucleus, where it specifically activates the transcription of plant genes (Kay *et al*., 2007; Marois *et al*., 2002). In resistant pepper plants, AvrBs3 activates the *Bs3* gene, thus inducing the HR (Römer *et al*., 2007). Another well‐characterized T3E from *Xcv* is AvrBsT, which is a member of the YopJ/AvrRxv family and acts as an acetyltransferase (Cheong *et al*., 2014). The catalytic activity of AvrBsT is required to suppress plant defence, e.g. the HR triggered by the effector AvrBs1 in pepper (Szczesny *et al*., 2010). On the other hand, AvrBsT is recognized in pepper and *Nicotiana benthamiana*, dependent on its catalytic activity (Escolar *et al*., 2001; Orth *et al*., 2000). XopB lacks conserved domains or known functional motifs, but contributes to disease, and, together with XopS, to bacterial growth *in planta*. Furthermore, XopB suppresses defence responses triggered by several T3Es, e.g. XopG (Schulze *et al*., 2012). In *N. benthamiana* and *Arabidopsis thaliana*, XopB expression elicits a cell death reaction, suggesting strong interference with plant metabolism/signalling or the induction of defence mechanisms in these plants (Priller *et al*., 2016; Schulze *et al*., 2012). For many T3Es from *Xcv*, however, a contribution to bacterial virulence is still enigmatic.

The T3S apparatus of *Xcv* consists of ring structures in both bacterial membranes surrounding the protein transportation conduit, a predicted cytoplasmic ring and the ATPase complex which fuels the secretion process (Büttner, 2012; Hartmann and Büttner, 2013; Hausner and Büttner, 2014; Lorenz and Büttner, 2009, 2011; Lorenz *et al*., 2012). This core apparatus is prolonged by an extracellular pilus whose assembly requires the predicted periplasmic inner rod protein HrpB2 and the pilus subunit HrpE (Hartmann *et al*., 2012; Weber *et al*., 2005). Finally, the translocator HrpF and the putative translocon component XopA are secreted by the T3S system and presumably insert into the plant plasma membrane forming a pore to facilitate the translocation of T3Es (Büttner *et al*., 2002; Noël *et al*., 2002). HrpB2, HrpE, HrpF and XopA are substrates of the T3S core apparatus, but are, under normal conditions, not translocated into the plant cell and are therefore termed non‐effectors (Büttner *et al*., 2004; Noël *et al*., 2002; Rossier *et al*., 2000).

The components of the *Xcv* T3S system are encoded by *hrp* (HR and pathogenicity) and *hrc* (*hrp* conserved) genes, whose expression is induced *in planta* and in special minimal media by an as yet unknown signal (Bonas *et al*., 1991; Schulte and Bonas, 1992). The signal activates HrpG, a member of the OmpR family of two‐component response regulators, which induces, in turn, the expression of the AraC‐type transcription activator gene *hrpX* (Schulte and Bonas, 1992; Wengelnik and Bonas, 1996; Wengelnik *et al*., 1996). HrpX binds to the PIP (plant‐inducible promoter) box, a *cis*‐regulatory element present in the promoters of most *hrp* operons and many T3E genes (Koebnik *et al*., 2006). The co‐expression of T3S system components and substrates is thought to require a tight post‐translational control to first assemble the T3S core system before switching to the secretion of extracellular components (non‐effectors) and, finally, to the translocation of T3Es (Büttner and He, 2009). Export control proteins of the *Xcv* T3S system are encoded by *hpa* (*hrp*‐associated) genes, which are not essential for pathogenicity, but contribute to *Xcv* growth and plant reactions (Büttner and He, 2009). HpaC is a substrate‐specificity‐switch protein, i.e. it suppresses the secretion of the early substrate HrpB2 and promotes the secretion of translocon and T3E proteins (Lorenz *et al*., 2008). The next switch involves the general T3S chaperone HpaB which promotes T3E secretion and inhibits the translocation of non‐effectors (Büttner *et al*., 2004). During the early stages of T3S, HpaB interacts with its regulator HpaA which, probably after full assembly of the T3S system, is translocated into the plant cell, thus liberating HpaB (Lorenz *et al*., 2008). HpaB promotes the secretion of all *Xcv* T3Es tested so far, albeit to different extents (Büttner *et al*., 2004, 2006; Scheibner *et al*., 2017; Schulze *et al*., 2012).

How T3Es are recognized by the T3S system is poorly understood. The non‐cleaved T3S signal is typically located within the N‐terminal 15–30 amino acids of T3Es. It appears to be taxonomically universal and conserved in plant‐ and animal‐pathogenic bacteria, but lacks a discernible amino acid consensus sequence (Arnold *et al*., 2009; Samudrala *et al*., 2009). However, N‐terminal T3E regions share distinct biophysical features and a bias for particular amino acids, e.g. serine (Ser) and proline (Pro) (Arnold *et al*., 2009; Guttman *et al*., 2002; McDermott *et al*., 2011; Wang *et al*., 2013). The resulting structural flexibility of the N‐terminal protein region and probably active unfolding allow the T3Es to pass the narrow inner channel of the T3S system (Galán *et al*., 2014). Although T3S signals are present in both T3Es and non‐effectors, only T3Es enter the plant cell under normal conditions. Therefore, it is assumed that effectors possess an additional signal for translocation. Notably, non‐effectors can be translocated by *Xcv* strains lacking the export control proteins HpaB and HpaC, respectively (Büttner *et al*., 2004; Scheibner *et al*., 2016). Studies in *Xcv* and *Erwinia amylovora*, using serial N‐terminal lengths of T3Es fused to translocation reporter proteins, located the putative translocation signal in N‐terminal protein regions downstream of the T3S signal (Mudgett *et al*., 2000; Oh *et al*., 2010; Scheibner *et al*., 2017). Based on sequence comparison of nine *Xcv* T3Es, a consensus translocation motif (TrM) has been proposed (Escolar *et al*., 2001), which served as a starting point for the present study. We introduced mutations into the putative TrMs in XopB and other T3Es from *Xcv*. We show that the arginine (Arg)‐rich TrM mediates XopB binding to cardiolipin (CL), a major lipid in *Xanthomonas* membranes, and indeed contributes to the secretion and translocation of XopB, AvrBs1 and AvrBsT by *Xcv*.

## RESULTS

### The N‐terminal region of XopB contains a TrM

**Figure 1:**
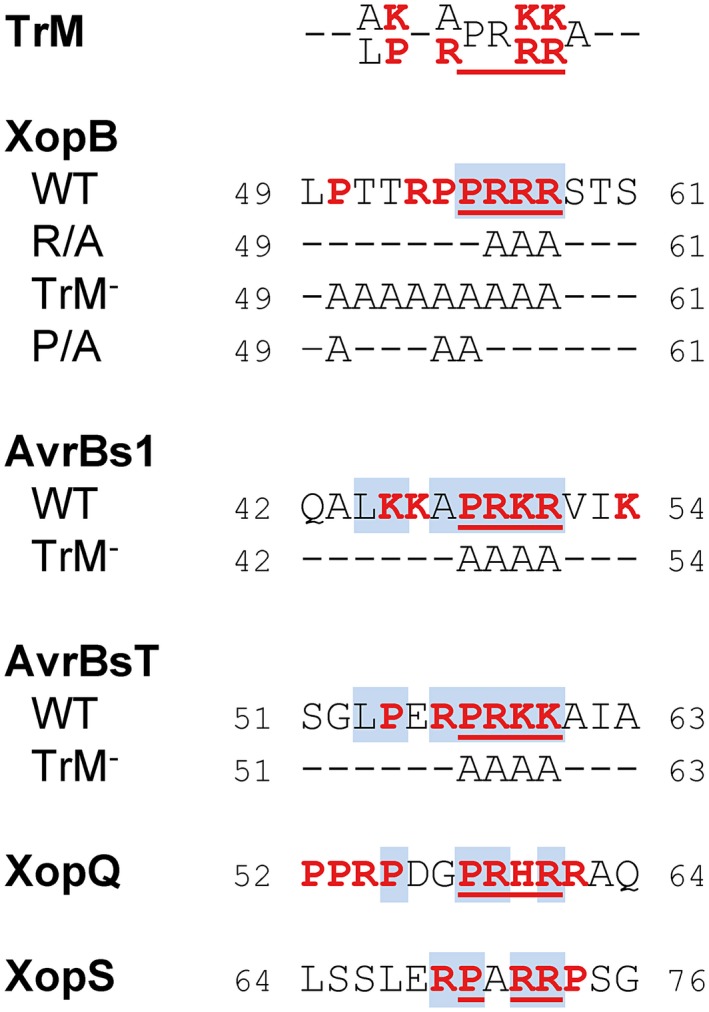
Putative type III translocation motifs in type III effectors (T3Es) from *Xanthomonas campestris* pv. *vesicatoria *(*Xcv*). The proposed translocation motif (TrM; Escolar *et al*., 2001) is compared with selected T3Es from *Xcv *and mutant derivatives tested in this study. Amino acids which match the TrM consensus sequence are highlighted in blue, and proline (Pro), arginine (Arg), lysine (Lys) and histidine (His) residues in red. The most conserved sequence consisting of Pro followed by three basic amino acids is underlined. WT, wild‐type. Amino acids are given in the one‐letter code. Numbers refer to amino acid positions. [Colour figure can be viewed at wileyonlinelibrary.com]

Previously, we proposed a TrM within the N‐terminal 150 amino acids of different *Xanthomonas* T3Es, the majority of which were still unknown at the time (Escolar *et al*., 2001; Fig. 1). We identified a similar motif in XopB (Fig. 1). In this motif, the most prominent feature of the Pro/Arg‐rich sequence is conserved, i.e. a Pro followed by three basic amino acids. Furthermore, the XopB motif contains an additional Arg and two Pro residues, although their positions differ from the proposed TrM consensus sequence (Fig. 1). To determine the functional relevance of the TrM, three different mutant derivatives of XopB were generated: (i) the Arg residues were substituted by alanine (R_56–58_A; R/A); (ii) the whole motif was replaced (P_50_‐R_58_A; TrM^–^); and (iii) all Pro residues in the TrM were exchanged (P_50,54,55_A; P/A).

**Figure 2:**
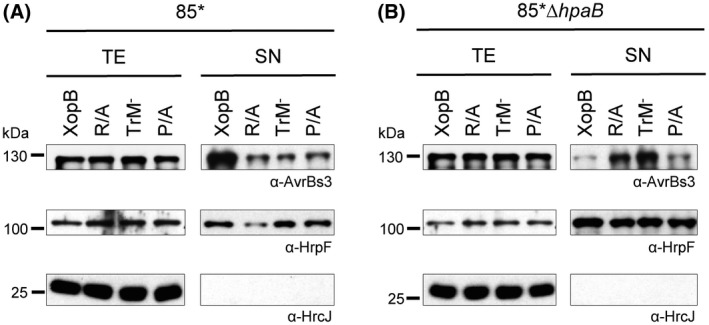
Type III secretion of XopB and derivatives. *Xcv* strains 85* (A) and 85*Δ*hpaB* (B) ectopically expressing XopB_1–177_::AvrBs3∆2 or TrM mutant derivatives with exchanges of arginine (R_56–58_A; R/A), the complete motif (P_50_‐R_58_A, TrM^–^) or proline (P_50,54,55_A; P/A) were grown in secretion medium. Equal amounts of total cell extracts (TE) and culture supernatants (SN) were analysed by immunoblot using AvrBs3‐, HrpF‐ and HrcJ‐specific antibodies. HrcJ served as lysis control.

**Figure 3:**
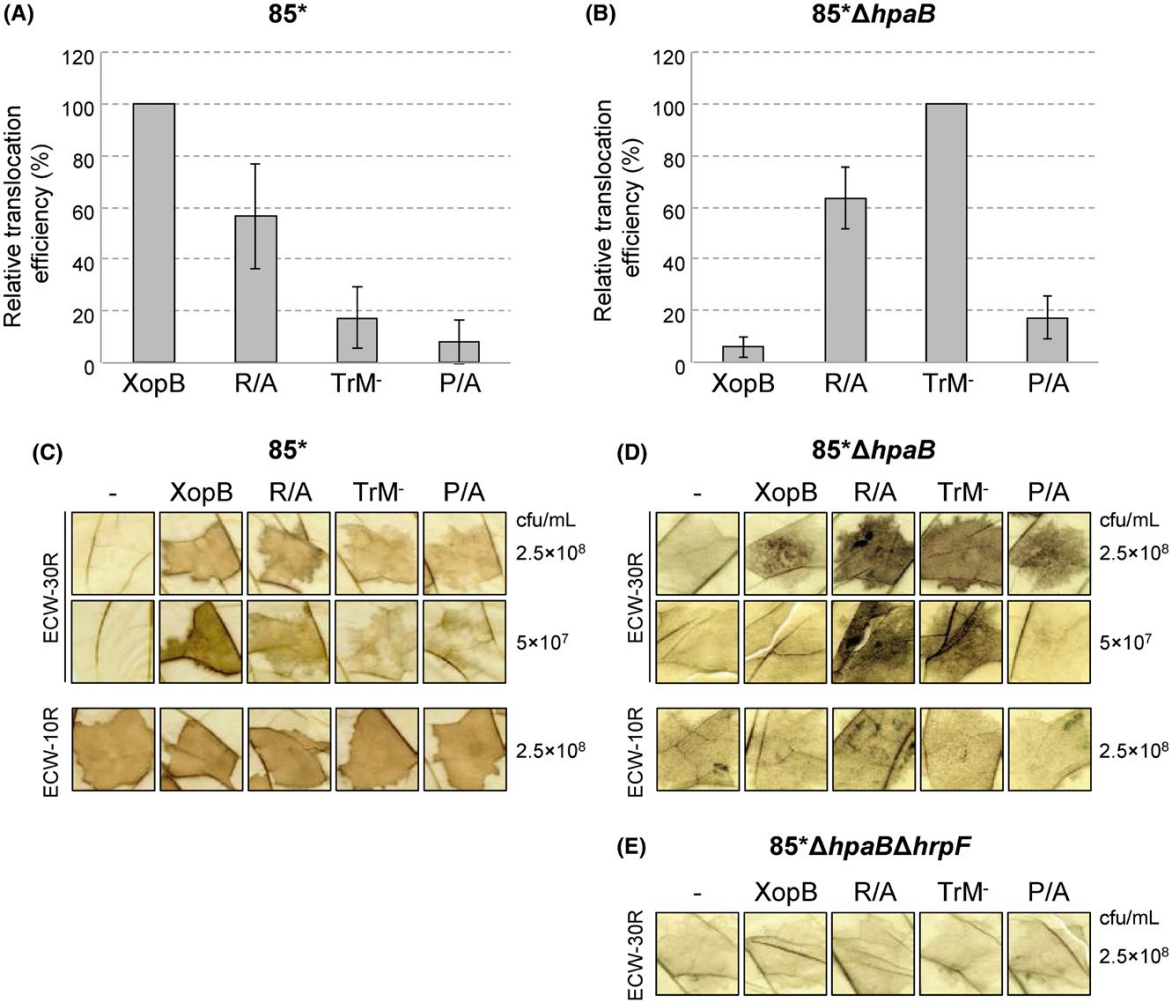
*In planta* translocation assay of XopB and derivatives.* Xcv* strains 85* (A) and 85*Δ*hpaB* (B) ectopically expressing XopB_1–177_::AvrBs3∆2 or TrM mutants were inoculated into leaves of AvrBs3‐responsive pepper ECW‐30R plants. At 3 days post‐inoculation (dpi), leaves were bleached in ethanol to better visualize the hypersensitive response (HR). HR intensities, indicative of the translocation efficiency of a particular fusion protein, were evaluated as described in Experimental Procedures. (A, B) Data represent the average of at least five independent experiments. The translocation efficiencies of XopB WT (A) and XopB TrM^–^ (B) were set to 100%. Error bars indicate standard deviation (SD). (C, D) Representative plant reactions of pepper ECW‐30R at 3 dpi of 85* (C) and 85*Δ*hpaB* derivatives (D) (top panels). As control for functional type III secretion (T3S), the same strains were inoculated into leaves of ECW‐10R pepper plants which recognize the T3E AvrBs1; reactions were documented at 3 dpi (bottom panels). (E) Reactions of ECW‐30R pepper plants at 3 dpi of 85*Δ*hpaB*Δ*hrpF *derivatives expressing the indicated proteins. The expression of AvrBs3∆2 fusion proteins is shown in Fig. S6A–C (see Supporting Information). cfu, colony‐forming units. [Colour figure can be viewed at wileyonlinelibrary.com]

To analyse the type III‐dependent transport of the XopB mutant variants, we tested secretion *in vitro* and translocation *in planta* by an HR‐based reporter assay. For this, we used a XopB derivative containing the N‐terminal 177 amino acids of the effector translationally fused to our established T3S reporter protein AvrBs3Δ2. The latter is a variant of the T3E AvrBs3 lacking T3S and translocation signals (Szurek *et al*., 2002). The constructs encoding XopB_1–177_::AvrBs3Δ2 and the respective R/A, TrM^–^ and P/A derivatives were introduced into *Xcv* strain 85*, which constitutively expresses the *hrp* genes as a result of a constitutively active HrpG variant (Wengelnik *et al*., 1999). *Xcv *85* ectopically expressing XopB_1–177_::AvrBs3Δ2 secretes XopB into the culture medium and triggers the AvrBs3‐dependent HR in pepper plants containing the *Bs3* resistance gene (Schulze *et al*., 2012). As shown in Fig. 2A, secretion of the R/A derivative was reduced compared with the wild‐type (WT) XopB‐reporter fusion. Furthermore, the strain triggered a delayed HR in *Bs3* pepper plants, indicative of a reduced translocation of XopB_1–177;R/A_::AvrBs3Δ2 [Fig. 3A,C; for scoring parameters, see Fig. S1 (Supporting Information) and Experimental Procedures]. The secretion and translocation efficiency of XopB_1–177;P/A_::AvrBs3Δ2 was even more reduced, and comparable with XopB_1–177;TrM_–::AvrBs3Δ2 (Figs 2A, 3A,C). A translocation assay with derivatives of the *Xcv* WT strain 85‐10 expressing XopB_1–177_::AvrBs3Δ2 and the mutant variants led to comparable results (Fig. S2A,B, see Supporting Information).

None of the fusion proteins caused a visible HR when expressed by a Δ*hrpF* mutant, which lacks the translocon gene, confirming that the observed phenotypes depend on a functional T3S system (Fig. S2C). *Agrobacterium*‐mediated expression of all fusion proteins in *Bs3* pepper plants induced an HR comparable with that of WT XopB_1–177_::AvrBs3Δ2 (Fig. S3, see Supporting Information). This indicates that the delayed HR induced by *Xcv* expressing TrM mutant derivatives is not a result of reduced activity of the reporter fusions, but caused by a reduced protein translocation into the plant cell. Both 85‐10 and 85* derivatives translocated AvrBs1 as the WT strain as suggested by the HR in *Bs1* pepper plants (Figs 3C, S2B). This suggests that the analysed XopB‐reporter fusions had no general inhibitory effect on T3S activity.

### The TrM determines the HpaB dependence of type III‐dependent XopB export

The T3S chaperone HpaB specifically promotes the secretion and translocation of T3Es and inhibits the translocation of non‐effectors (Büttner *et al*., 2004; Scheibner *et al*., 2017; Schulze *et al*., 2012). Using a Δ*hpaB *mutant strain, we tested whether the TrM determines the HpaB dependence of XopB translocation. Notably, XopB_1–177;R/A_ was more efficiently secreted and, in our HR‐based reporter assay, translocated by *Xcv* strain 85*Δ*hpaB* than the XopB WT fusion (Figs 2B, 3B,D). The TrM^–^ derivative was even better translocated than XopB_1–177;R/A_::AvrBs3Δ2. By contrast, XopB_1–177;P/A_::AvrBs3Δ2 was secreted and translocated only in small amounts, i.e. comparable with the XopB WT fusion (Figs 2B, 3B,D). Translocation assays with a Δ*hpaB*Δ*hrpF* double mutant confirmed the dependence of the observed phenotypes on a functional T3S apparatus (Fig. 3E). Taken together, XopB derivatives lacking Arg residues in the TrM are reminiscent of non‐effectors with respect to HpaB dependence, i.e. translocation of XopB_1–177;R/A_ and XopB_1–177;TrM_– by the *Xcv* WT strain was strongly reduced, whereas they were well translocated by the *hpaB *mutant. By contrast, the exchange of only Pro residues did not alter the translocation efficiency in the absence of HpaB.

To elucidate whether HpaB might control XopB export by direct protein–protein interaction via the TrM, we performed glutathione‐*S*‐transferase (GST) pull‐down assays. As shown in Fig. S4 (see Supporting Information), XopB and HpaB indeed interacted *in vitro*. However, mutations in the TrM of XopB did not affect this interaction. Furthermore, the 41‐amino‐acid XopB region encompassing the TrM was not bound by HpaB, suggesting that the TrM does not correspond to the HpaB binding site (Fig. S4).

### The TrM is dispensable for XopB activity *in planta*

To determine whether the TrM is solely required for type III‐dependent XopB export from *Xcv* or if it contributes to XopB function *in planta*, we expressed WT XopB, an N‐terminally truncated variant lacking the TrM (XopB_Δ2–99_) and the XopB_R/A_ derivative using agroinfection. First, we scored for XopB‐dependent cell death elicitation in leaves of *N. benthamiana* (Schulze *et al*., 2012). The XopB derivative lacking the N‐terminal 99 amino acids (XopB_Δ2–99_) induced cell death in *N. benthamiana*, albeit weaker than the WT protein (Fig. S5A, see Supporting Information). This phenotype might be a result of lower protein accumulation. By contrast, R‐to‐A substitutions in the TrM had no effect on the cell death‐inducing activity of XopB (Fig. S5A).

The second XopB activity tested was its ability to suppress the XopG‐dependent HR in *Nicotiana tabacum *(Schulze *et al*., 2012). Interestingly, XopB_Δ2–99_ allowed the induction of XopG‐triggered cell death, whereas XopB_R/A_ efficiently suppressed XopG HR (Fig. S5B). This suggests that missing protein regions located within the first 99 amino acids, but unrelated to the TrM, are responsible for the loss of XopB_Δ2–99_ function. Taken together, our results point to type III‐dependent transport as the primary function of the TrM in XopB.

### XopB associates with the bacterial membrane

**Figure 4:**
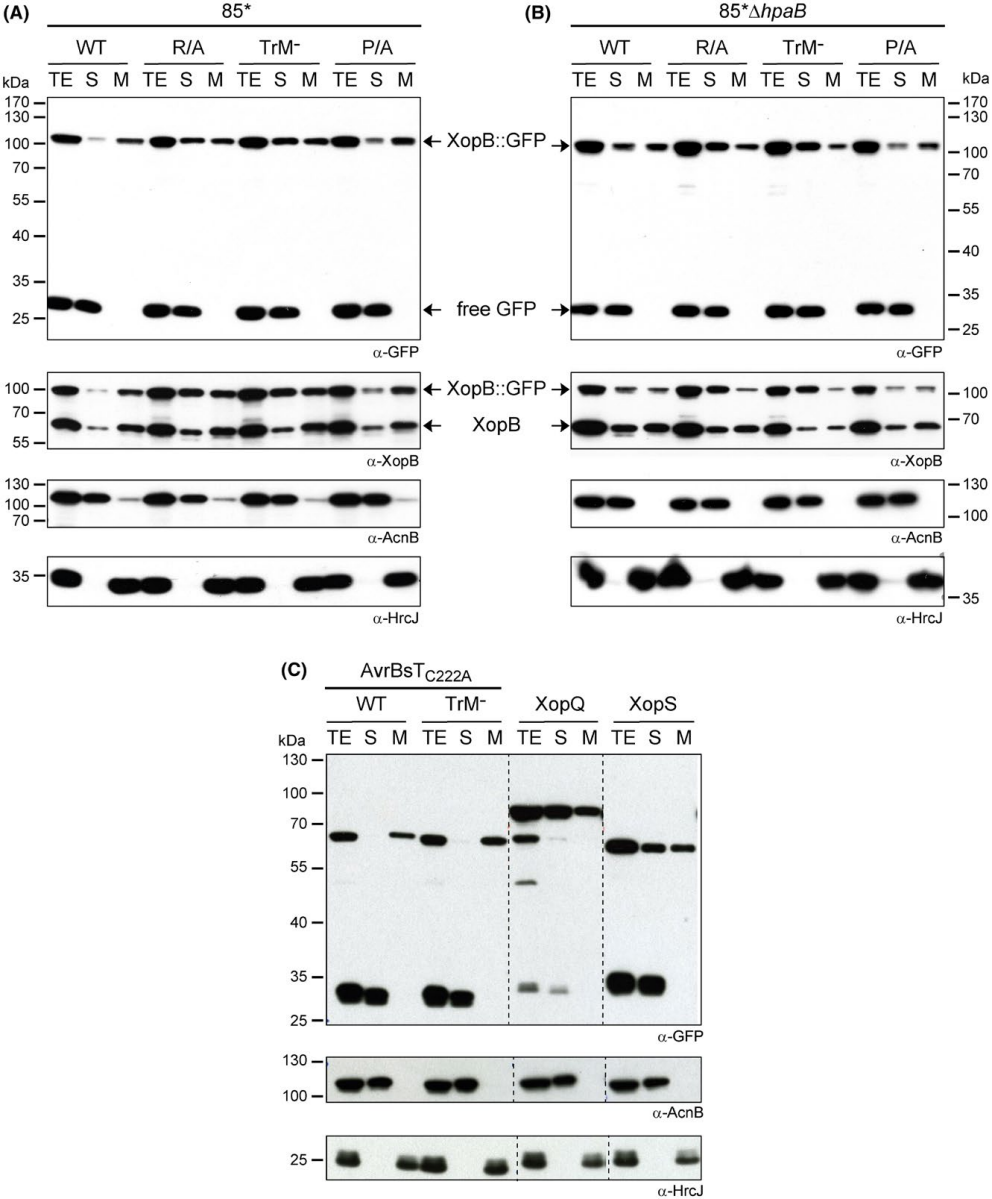
Subcellular localization of XopB and other selected T3Es in *Xcv. *To test for a possible membrane association of XopB and other T3Es, fractionation studies were performed. (A, B) *Xcv *strains 85* (A) and 85*Δ*hpaB* (B) ectopically expressing XopB WT and TrM mutant derivatives. (C) Strain 85* ectopically expressing AvrBsT, XopQ and XopS. Bacteria were grown in MA medium (pH 7.0). All proteins were expressed as C‐terminally green fluorescent protein (GFP)‐tagged derivatives. Total protein extracts (TE), membrane (M) and soluble (S) fractions were analysed by immunoblotting using GFP‐ and XopB‐specific antibodies. Antibodies against soluble aconitase B (AcnB) and membrane‐bound HrcJ served as controls. [Colour figure can be viewed at wileyonlinelibrary.com]

How could the Pro/Arg‐rich TrM promote XopB export? T3Es are probably transported in an at least partially unfolded state, which is achieved by interaction with the ATPase of the T3S system (Akeda and Galán, 2005; Galán *et al*., 2014; Lorenz and Büttner, 2009). This might be supported by the interaction with negatively charged membrane surfaces, which can also promote partial protein unfolding (Musatov and Sedlák, 2017). *Xanthomonas* membranes are rich in CL (Aktas and Narberhaus, 2015), a dimeric phospholipid potentially carrying two negative charges. Notably, CL binding of proteins is often attributed to regions rich in basic amino acids [Arg, histidine (His) and lysine (Lys)], which form a phosphate‐binding patch (Musatov and Sedlák, 2017; Planas‐Iglesias *et al*., 2015). To determine whether XopB localizes to membranes in *Xcv*, we performed fractionation experiments with cells grown in MA medium (pH 7), which allows expression of the T3S system and T3Es, but no secretion. To discriminate between endogenous XopB protein and mutant derivatives, the latter were 3′‐translationally fused to the green fluorescent protein (GFP). GFP‐tagged XopB protein was detected in both soluble and membrane fractions of *Xcv* (Fig. 4A). Substitution of the Arg residues or the whole TrM appeared to result in more soluble XopB::GFP protein, whereas the distribution of XopB_P/A_::GFP was comparable with that of the WT fusion. As an internal control, we analysed the localization pattern of endogenous XopB using a XopB‐specific antibody. As shown in Fig. 4A, the subcellular distribution of endogenous XopB in *Xcv* was not altered by ectopic expression of the different XopB::GFP derivatives.

Previous studies have revealed direct interactions of *Xanthomonas* T3Es with the T3S chaperone HpaB (Büttner *et al*., 2004), which is also membrane associated (Lorenz and Büttner, 2009). We wondered whether HpaB might contribute to the interaction of XopB with the bacterial membrane. However, fractionation studies revealed no reproducible difference in the localization pattern of XopB::GFP or the mutant derivatives in the Δ*hpaB* strain (Fig. 4B), which argues against a role of HpaB in the membrane association of XopB.

### XopB binds CL dependent on the TrM

**Figure 5:**
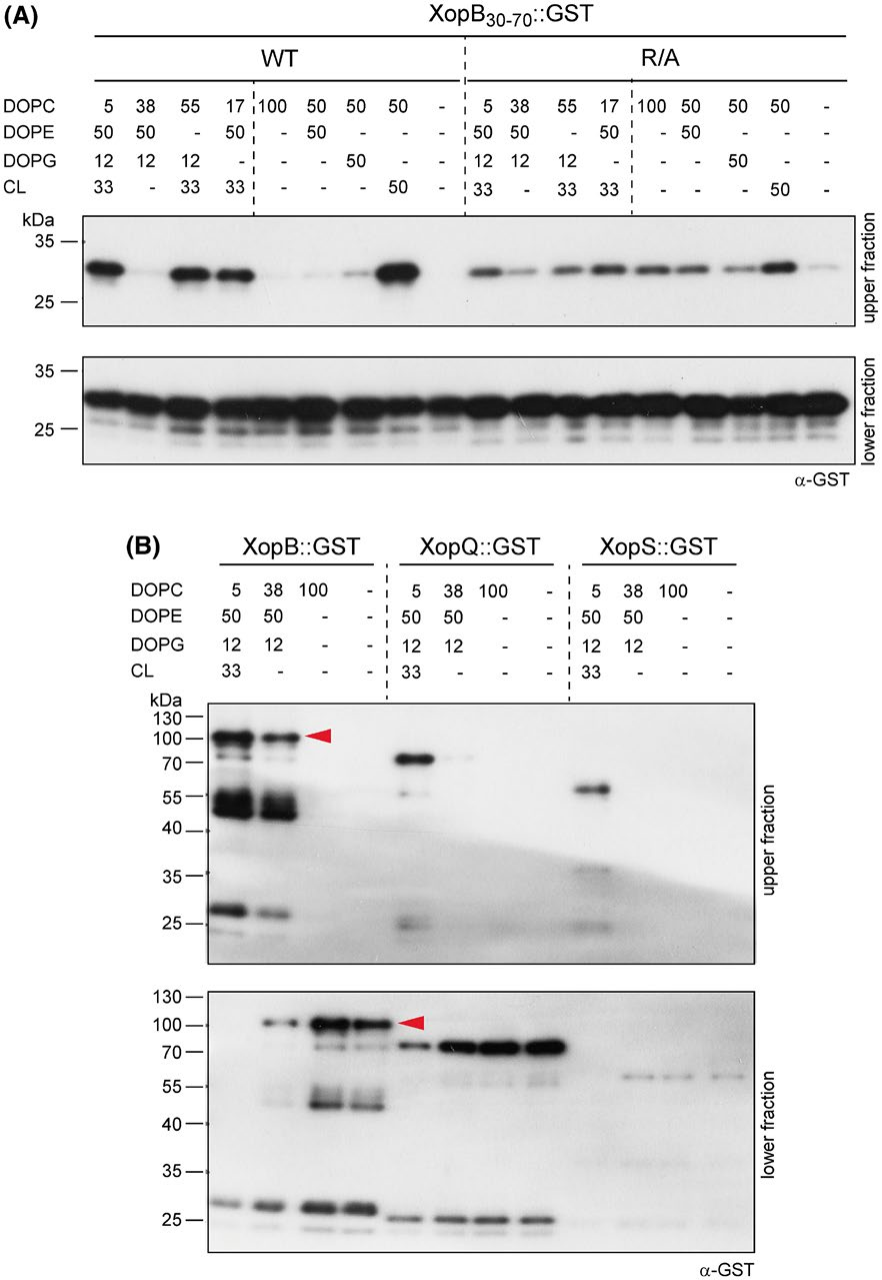
TrM‐dependent lipid binding. (A) Purified XopB_30–70_::GST protein and a respective R/A derivative (Fig. S7, see Supporting Information) were incubated with liposomes of the indicated lipid composition (in mol.%) and analysed by sucrose gradients. The upper and lower fractions containing liposome‐bound and free protein, respectively, were analysed by immunoblot using a glutathione‐*S*‐transferase (GST)‐specific antibody. (B) Liposome flotation assay with purified XopB, XopQ and XopS fused to GST. Arrowheads mark the XopB full‐length protein. CL, cardiolipin; DOPC, 1,2‐dioleoyl‐*sn*‐glycero‐3‐phosphocholine; DOPE, 1,2‐dioleoyl‐*sn*‐glycero‐3‐phosphoethanolamine; DOPG, 1,2‐dioleoyl‐*sn*‐glycero‐3‐phospho‐1′‐*rac*‐glycerol; WT, wild‐type. [Colour figure can be viewed at wileyonlinelibrary.com]

To investigate whether XopB attaches to the *Xcv* membrane via interaction with membrane lipids, we analysed the lipid‐binding potential of XopB by liposome flotation assays. For this, a 41‐amino‐acid polypeptide encompassing the TrM (XopB_30–70_) was 3′‐translationally fused to GST. This protein version was much more stable and easier to purify than the XopB full‐length protein (see below). Unilamellar vesicles of defined size and lipid composition were incubated with XopB::GST and XopB_R/A_::GST proteins, respectively, at the bottom of a sucrose gradient. Ultracentrifugation resulted in vesicle‐bound proteins floating in the upper fraction of the gradient, whereas unbound proteins remained in the lower fraction. First, we used liposomes similar to the standard lipid composition of *Xanthomonas* membranes, which mainly consist of ~50% phosphatidylethanolamine (PE), ~33% CL, ~13% phosphatidylglycerol (PG) and ~6% phosphatidylcholine (PC) (Aktas and Narberhaus, 2015). As shown in Fig. 5A, the XopB_30–70_::GST fusion protein bound to liposomes, and substitution of the Arg residues in the TrM drastically reduced liposome binding.

To identify specific membrane components bound by the TrM, different lipid compositions were tested. On the one hand, particular membrane lipids of the *Xanthomonas* standard mixture were exchanged by PC. On the other, the lipids were tested individually, always in a one‐to‐one ratio with PC to facilitate the formation of adequate liposomes. As shown in Fig. 5A, the XopB fusion protein preferentially bound to CL‐containing liposomes.

### Analysis of other *Xcv* T3Es

**Figure 6:**
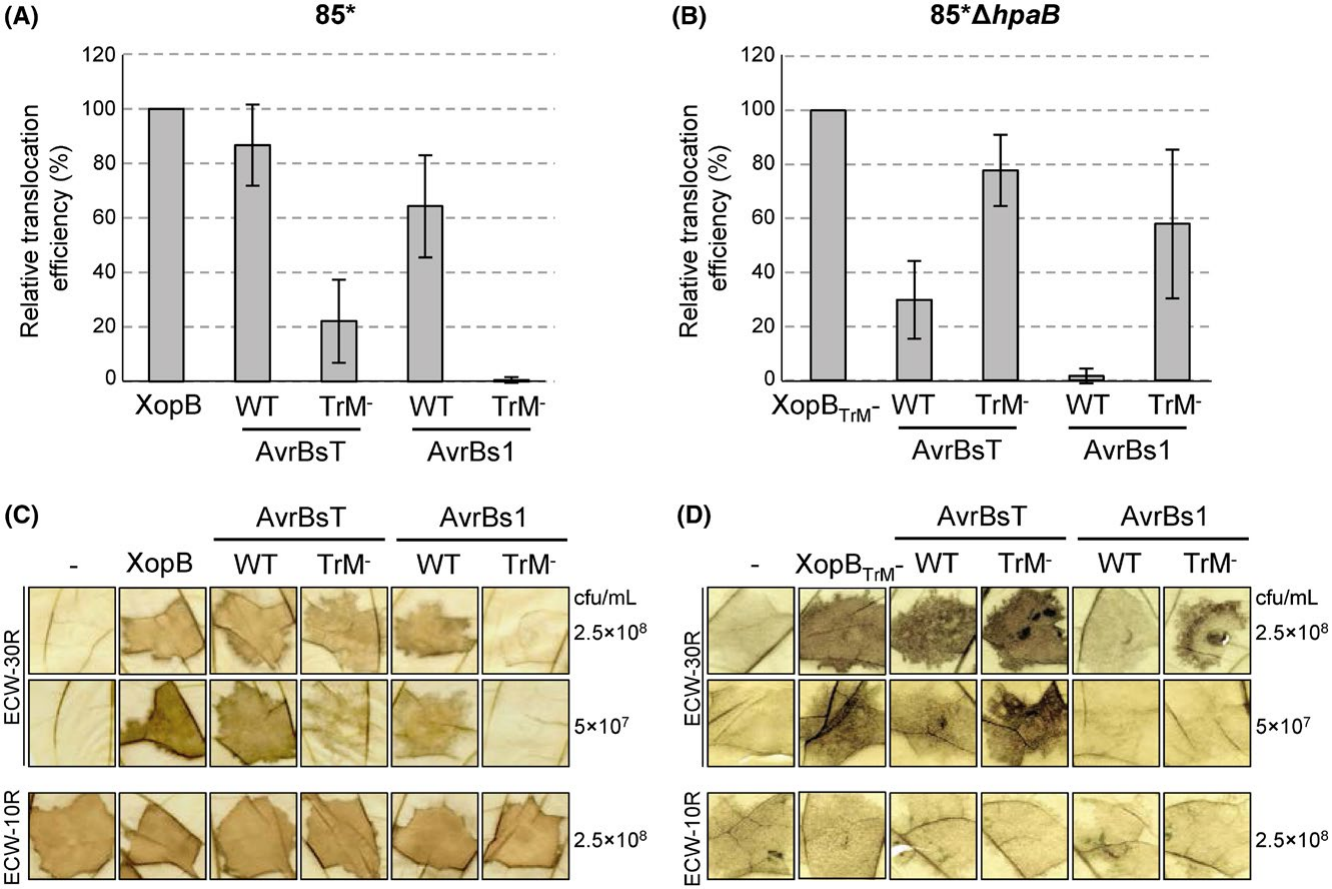
*In planta* translocation assay of AvrBs1, AvrBsT and derivatives. *Xcv* strains 85* (A) and 85*Δ*hpaB* (B) ectopically expressing AvrBs1_1–111_::AvrBs3Δ2, AvrBsT_C222A_::AvrBs3Δ2 and the respective TrM^–^ mutants (AvrBs1_1–111;P48‐R51A_::AvrBs3Δ2, AvrBsT_C222A;P57‐K60A_::AvrBs3Δ2) were inoculated into leaves of pepper ECW‐30R plants. Leaves were bleached in ethanol at 3 dpi. The intensity of plant reactions, indicative of the translocation efficiency of the respective T3E fusion proteins, was evaluated as described in Experimental Procedures. The translocation efficiency of XopB WT (A) and XopB TrM^–^ (B) was set to 100%. Data represent the average of at least five independent experiments; error bars indicate SD. (C, D) Representative plant reactions of pepper ECW‐30R at 3 dpi of 85* (C) and 85*Δ*hpaB* derivatives (D) (top panels). Expression of AvrBs3∆2 fusion proteins is shown in Fig. S6A,B. As control for functional T3S, the same strains were inoculated into leaves of AvrBs1‐responsive ECW‐10R pepper plants and reactions were analysed at 3 dpi (bottom panels). cfu, colony‐forming units. [Colour figure can be viewed at wileyonlinelibrary.com]

To investigate whether the results obtained for XopB apply to other T3Es from *Xcv*, we tested derivatives of AvrBs1 and AvrBsT and respective TrM mutants in our *in planta* translocation assay. As WT AvrBsT induces a slow cell death reaction in all pepper lines (Minsavage *et al*., 1990), which might interfere with the HR‐based translocation assay, we used an enzymatically inactive AvrBsT version (C_222_A) for fusions to the AvrBs3Δ2 reporter. As shown in Fig. 6A and C, *Xcv* strains expressing AvrBs1_1–111_::AvrBs3Δ2 or AvrBsT_C222A_::AvrBs3Δ2 elicited a strong HR in *Bs3* pepper plants, whereas strains expressing the respective TrM mutants (AvrBs1_1–111;P48‐R51A_::AvrBs3Δ2, AvrBsT_C222A;P57‐K60A_::AvrBs3Δ2) caused delayed cell death reactions. This suggests that the TrM promotes type III‐dependent translocation of both T3E‐reporter fusions. When the AvrBs1 and AvrBsT fusions were expressed by *Xcv* strain 85*Δ*hpaB*, the TrM^–^ mutants caused a faster HR, i.e. were more efficiently translocated, compared with the WT proteins (Fig. 6B,D), similar to the results obtained for XopB. HR‐inducing activity of the fusion proteins in *Bs3* pepper plants was confirmed by *Agrobacterium*‐mediated expression (Fig. S3). Taken together, the TrM increases the efficiency of T3E translocation in *Xcv *and determines HpaB dependence, at least in the case of XopB, AvrBs1 and AvrBsT.

To analyse potential membrane binding of the effectors, fractionation analyses were performed with *Xcv* 85* expressing GFP‐tagged AvrBsT and the TrM^–^ derivative. As shown in Fig. 4C, AvrBsT::GFP was almost exclusively detected in the membrane fraction. Mutation of the TrM had no obvious effect on the localization pattern. AvrBs1::GFP was highly unstable when expressed in *Xcv* 85*, and was therefore not analysed. Instead, we tested GFP‐tagged XopQ and XopS, both of which also contain TrM‐like Pro/Arg‐rich sequence stretches in their N‐terminal regions (Fig. 1). Both proteins were found in the *Xanthomonas* membrane and soluble fractions (Fig. 4C), as observed for XopB. This suggests that at least partial association with the bacterial membrane might be a general feature of T3Es from *Xcv.*

To investigate whether, and, if so, which, membrane lipids are bound by the T3Es, we tested full‐length T3E‐GST fusion proteins in liposome flotation assays. For technical reasons, AvrBs1 and AvrBsT could not be analysed because the purification of recombinant proteins failed. Therefore, we tested GST fusions of XopQ and XopS. In addition, we included full‐length XopB::GST, although this protein was less stable. As shown in Fig. 5B, XopB, XopQ and XopS fusion proteins bound the liposomes dependent on CL. Unexpectedly, the XopB::GST protein showed considerable residual affinity to liposomes even in the absence of CL. Given the strong CL preference of the 41‐amino‐acid XopB fragment (Fig. 5A), this suggests the presence of an additional lipid binding site in XopB with different specificity. Taken together, our analyses suggest that different *Xcv* T3Es bind the bacterial membrane using CL as preferential docking site.

## DISCUSSION

T3S is essential for the pathogenicity of most Gram‐negative phytopathogenic bacteria, but is still barely understood. Here, we demonstrate that several T3Es from *Xcv *contain a conserved Pro/Arg‐rich motif, termed TrM, which promotes type III‐dependent secretion and translocation. Our studies suggest that the Arg residues in the TrM contribute to the membrane binding of XopB, presumably by interaction with CL, one of the major lipids in *Xanthomonas* membranes. CL is involved in the function and stabilization of integral membrane proteins in bacteria and mitochondria (Musatov and Sedlák, 2017). Notably, CL is important for conformation, activity and localization of the Sec protein translocation machinery in *Escherichia coli* (Gold *et al*., 2010) and the function of other energy‐dependent transport systems, e.g. RND (resistance, nodulation, cell division) efflux pumps and Mg^2+^ transporters in *Pseudomonas putida* and *E. coli*, respectively (Bernal *et al*., 2007; Subramani *et al*., 2016). It is conceivable that CL also supports the assembly and/or activity of the T3S apparatus, which is an interesting subject for future studies. XopB might be recruited for type III‐dependent transport by binding to CL‐rich membrane regions around the T3S complexes. In addition, CL binding might promote unfolding of XopB, increasing its secretability.

In contrast with the Arg residues, substitution of Pro in the TrM had no obvious effect on the subcellular localization of XopB in *Xcv*. Instead, the Pro residues might increase the structural instability of the N‐terminal protein region to hold XopB in a partially unfolded, and thus secretion‐competent, state. In addition to a general structure‐dissolving function, the Pro residues in the TrM might help XopB dissociation from the membrane. This could explain why translocation of the XopB_P/A_ mutant, which still contains the CL‐binding Arg residues, was strongly reduced, whereas the XopB TrM^–^ mutant lacking both Arg and Pro was efficiently translocated by *Xcv *strain 85*Δ*hpaB*. T3E association to and dissociation from the bacterial membrane might, on the one hand, promote a close proximity between XopB and the membrane‐spanning T3S apparatus and a partial unfolding of the effector protein and, on the other, allow XopB release from the membrane for secretion.

**Figure 7:**
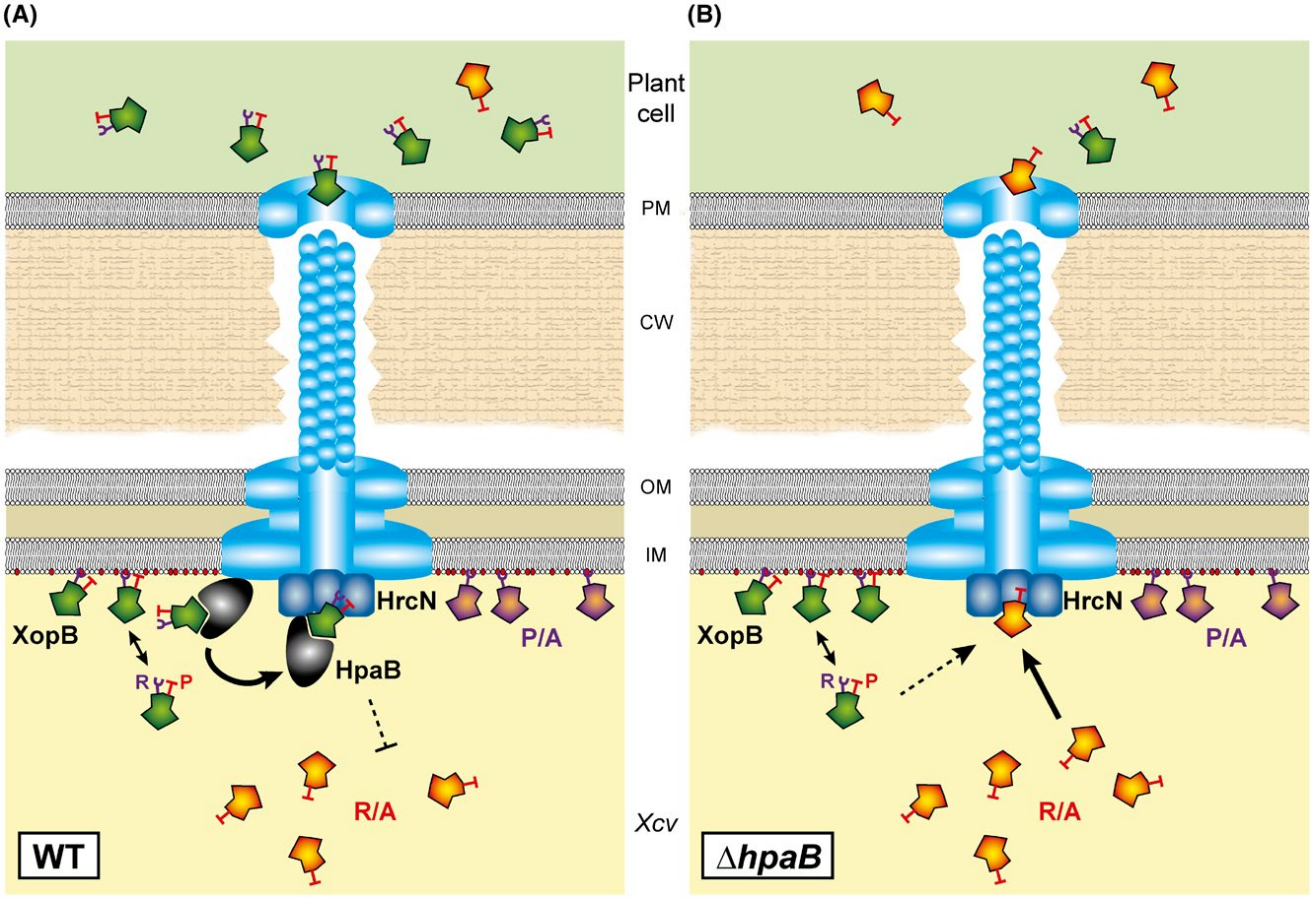
Putative role of the TrM in type III‐dependent XopB export by *Xcv*. Model of XopB secretion by an *Xcv* WT strain (A) and the *hpaB* mutant (B). The Arg residues (R) in the TrM of XopB facilitate interaction with CL in the inner membrane (red dots); Pro residues (P) promote XopB dissociation from the membrane. In the WT strain, HpaB promotes type III secretion (T3S) of membrane‐associated XopB by escorting it to the ATPase of the T3S system, HrcN (dark blue). HrcN dissociates the HpaB–XopB complex, liberating HpaB and energizes XopB unfolding and transport. HpaB might inhibit the secretion of free XopB or respective mutants (R/A). If HpaB is missing (B), free XopB proteins are preferentially secreted. Variants with substituted Pro residues stay at the membrane independent of HpaB. CW, cell wall; IM, inner membrane; OM, outer membrane; PM, plasma membrane. [Colour figure can be viewed at wileyonlinelibrary.com]

What is the role of the T3S chaperone HpaB in XopB export? In *hpaB* deletion mutants, XopB derivatives mutated in the Arg residues or the whole TrM were preferentially translocated, similar to the non‐effectors XopA and HrpF (Büttner *et al*., 2004). By contrast, translocation of these XopB variants by the WT strain, i.e. in the presence of HpaB, was strongly reduced, which is also reminiscent of non‐effectors. GST pull‐down assays showed that the reduced translocation was not caused by an inhibited XopB–HpaB interaction. It has been reported previously that the ability of T3S substrates to interact with HpaB *in vitro* is not sufficient for their translocation (Scheibner *et al*., 2017). XopB variants with mutations in Pro residues only were not well translocated by 85* or 85*Δ*hpaB*. Although a recent study proposed separate export signals for HpaB‐dependent and HpaB‐independent transport (Scheibner *et al*., 2017), we favour a spatio‐temporal model. According to this model (Fig. 7), HpaB in *Xcv* preferentially interacts with membrane‐associated XopB and recruits it to the T3S system. While HpaB is inhibited by binding to its regulator HpaA, i.e. during assembly of the T3S core apparatus and secretion of pilus and translocon components, XopB is ‘parked’ at the membrane. After complete assembly of the T3S system, HpaA is secreted and translocated into the plant cell, liberating HpaB (Lorenz *et al*., 2008). Now activated, HpaB ‘plucks’ XopB from the membrane and escorts it to the T3S apparatus, where the ATPase HrcN dissociates the HpaB–XopB complex, facilitating XopB secretion and the release of HpaB (Lorenz and Büttner, 2009). Soluble XopB derivatives, e.g. TrM mutants without Arg residues, might be bound only infrequently by the membrane‐associated HpaB, and therefore be less well secreted. It is also conceivable that HpaB inhibits the secretion of these XopB variants, similar to non‐effectors.

**Figure 8:**
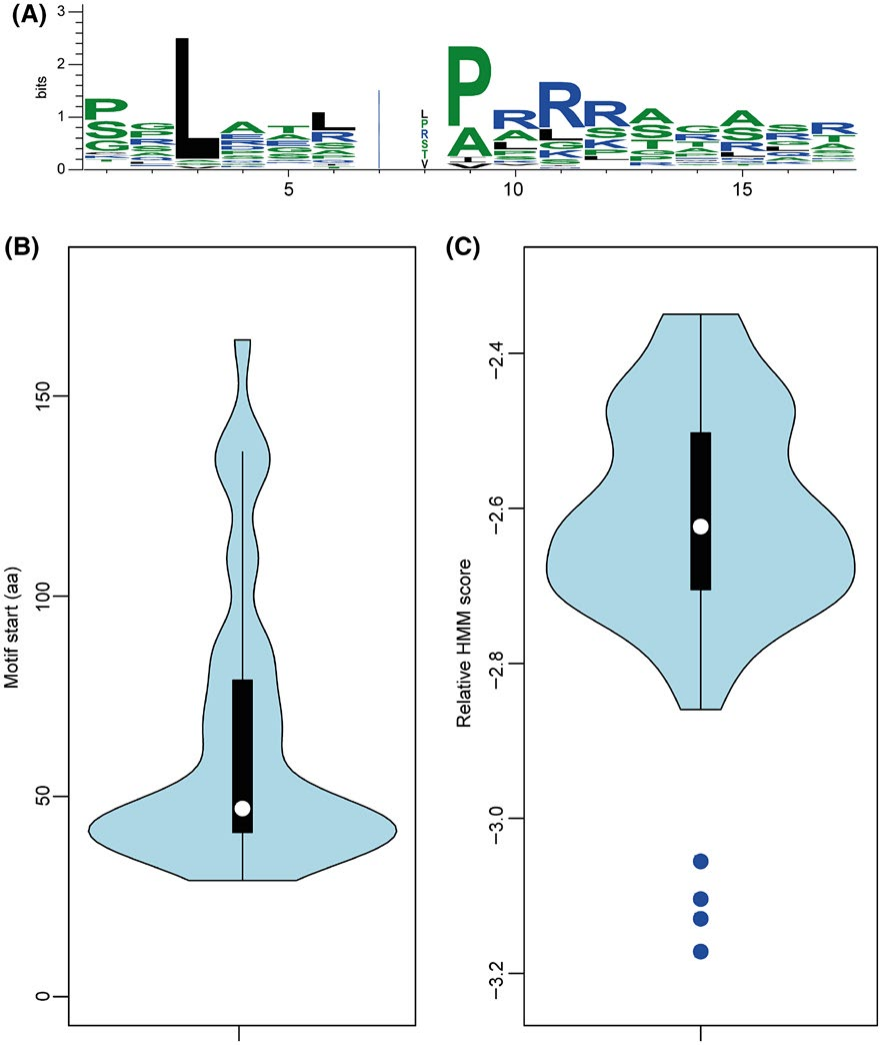
TrM consensus in T3Es from *Xcv. *(A) Sequence logo based on aligned motif occurrences predicted in the N‐terminal 180 amino acids of all 36 known T3Es from *Xcv* strain 85‐10 and of AvrBs3, AvrBs4, AvrBsT, AvrXv3 and AvrXv4 (see Table S1). Stack widths are scaled by the fraction of gaps in the alignment. Amino acids are coloured according to their hydrophobicity (blue, hydrophilic; green, neutral; black, hydrophobic). (B) Motif start sites. Violin plot of the positions of the first amino acid of motif matches counted from the N‐terminus. The black box represents the interquartile range with a white dot indicating the location of the median. The kernel density plot (blue), i.e. a smoothed histogram, of the motif starts was computed using a Gaussian kernel with a standard deviation of 7. (C) Relative hidden Markov model (HMM) scores of the best matches to the motif model in 41 T3E sequences. The black box represents the interquartile range with a white dot indicating the location of the median score. The kernel density plot (blue), i.e. a smoothed histogram, of the relative HMM scores was computed using a Gaussian kernel with a standard deviation of 0.04. Blue dots indicate the corresponding scores of the four non‐effectors XopB2, HrpE, HrpF and XopA. HMM scores are listed in Table S1. [Colour figure can be viewed at wileyonlinelibrary.com]

Can the proposed role of the TrM in T3S be generalized? Notably, the results for XopB apply to AvrBsT, AvrBs1, XopQ and XopS. We therefore assume that the TrM is a functional motif common to *Xcv* T3Es. We therefore analysed the N‐terminal 180 amino acids of 41 *Xcv *T3Es using a custom motif discovery approach based on profile hidden Markov models (HMMs; see Experimental Procedures). This revealed a refined TrM consensus (Fig. 8A; Table S1, see Supporting Information) with a length of 15–17 amino acids which, with the exception of AvrBs3, AvrBs4 and XopQ, largely corresponds to the TrMs identified manually by Escolar et al. (2001) and in this study (Fig. 1). Notably, the refined TrM not only contains a Pro/Arg‐rich stretch, but also a prominent leucine at position three (Fig. 8A). Future mutation studies of the prolonged motif and, in particular, the leucine residue might uncover an even stronger contribution to translocation efficiency than described here. In most cases, the refined TrM is located within the N‐terminal 65 amino acids of the T3Es but, importantly, not within amino acids 1–28 (Fig. 8B). This suggests that there is no overlap of the TrM with the T3S signal that is typically located within the N‐terminal 15–30 amino acids of a T3E and in which leucine is under‐represented (Arnold *et al*., 2009; Samudrala *et al*., 2009). Notably, when we used the motif discovery tool to predict TrM‐like sequences in type III‐secreted non‐effectors, the relative HMM scores (see Experimental Procedures) for the identified motifs were substantially lower than the scores for the predicted TrMs in T3Es (Fig. 8C; Table S1). Thus, it appears that HrpB2, HrpE, HrpF and XopA miss a true TrM, supporting the hypothesis that the TrM represents the discriminating sorting signal between T3Es and non‐effectors.

Taken together, our study experimentally confirms a TrM in several *Xcv* T3Es, and is a starting point for further analysis of non‐proteinaceous factors, such as CL, for their contribution to T3S.

## EXPERIMENTAL PROCEDURES

### Bacterial strains and growth conditions

*Escherichia coli *cells were grown at 37 °C in lysogeny broth (LB) medium (Bertani, 1951), and *Agrobacterium tumefaciens *and *Xcv* were grown at 30 °C in yeast extract broth (YEB) and nutrient yeast extract glycerol (NYG) medium (Daniels *et al*., 1984), respectively, supplemented with appropriate antibiotics. Plasmids were introduced into *E. coli* and *A. tumefaciens* by electroporation, and into *Xcv *by electroporation or conjugation, using pRK2013 as helper plasmid in triparental matings (Figurski and Helinski, 1979). Bacterial strains are listed in Table S2 (see Supporting Information).

### Plant material and inoculations

*Nicotiana benthamiana*, *N. tabacum *and pepper [*Capsicum annuum*; cultivars ECW‐30R (*Bs3*) and ECW‐10R (*Bs1*) (Minsavage *et al*., 1990)] plants were grown in a glasshouse under standard conditions (day and night temperatures of 23 °C and 19 °C, respectively, for *Nicotiana *spp., and 25 °C and 19 °C, respectively, for pepper, with 16 h of light and 40%–60% humidity). Mature leaves of 5–7‐week‐old *Nicotiana* spp. plants were inoculated with *A. tumefaciens *adjusted to an optical density at 600 nm (OD_600_) of 0.8 in infiltration medium (10 mm MES, pH 5.5, 10 mm MgCl_2_, 150 µm acetosyringone) using a needleless syringe. *Xcv *suspensions in 10 mm MgCl_2_ [2.5 × 10^8^ or 5 × 10^7^ colony‐forming units (cfu)/mL] were inoculated into leaves of 6‐week‐old pepper plants using a needleless syringe.

### Generation of Golden Gate vectors

The binary vector pGGA3 contains the backbone of pBGWFS7 (Karimi *et al*., 2005) and allows the expression of genes 3′‐translationally fused to a FLAG epitope under the control of the *35S* promoter. The *E. coli* expression vectors pGGE2, pGGE7 and pGGE9 contain the backbone of pQE60 (Qiagen, Hilden, Germany) in which the selectable marker (*ampR*) was exchanged by a kanamycin resistance cassette. Genes are expressed from the *T7* promoter. pGGE2 allows the expression of genes 5′‐translationally fused to GST. In pGGE7 and pGGE9, expressed genes are 3′‐translationally fused to a StrepII epitope (pGGE7) or to GST, which is separated from the protein of interest by a *Tobacco etch virus* (TEV) protease site (pGGE9). The broad host range vector pGGX7 contains the backbone of pBBR1MCS‐5 (Kovach *et al*., 1995) and allows the expression of genes 3′‐translationally fused to GFP under the control of the *lac* promoter. All vectors contain the chloramphenicol resistance *ccdB* selection cassette from pGWB2 (Nakagawa *et al*., 2007) flanked by *Bsa*I sites to allow Golden Gate cloning of DNA fragments, i.e. *Bsa*I⁄T4‐ligase cut‐ligation (Engler *et al*., 2008). Additional *Bsa*I restriction sites were removed during vector construction. Cloning details are available on request. To generate pGGA3_356, a *lacZ* selection marker and modules encoding N‐terminal, central and C‐terminal regions of AvrBs3Δ2 were cloned into pGGA3 by Golden Gate cloning. All vectors are listed in Table S2.

### Generation of T3E expression constructs and mutagenesis

The coding sequences of *xopB*, *avrBs1*, *xopQ* and *xopS* were amplified by polymerase chain reaction (PCR) from the genomic DNA of *Xcv* strain 85‐10 using Phusion polymerase (New England Biolabs GmbH, Frankfurt/Main, Germany), thereby introducing flanking *Bsa*I sites, and cloned into pJET1.2blunt. Internal *Bsa*I sites in *xopB* and *xopQ* were removed by splicing by overlap extension (SOE)‐PCR. pJET:xopB_R56–58A__ns was generated by SOE‐PCR using pJET:xopB_ns as a template. Other TrM mutations in *xopB*, *avrBs1* and *avrBsT *were generated by iterative site‐directed mutagenesis of pJET:xopB_ns, pJET:avrBs1_ns and pENTR/DavrBsT_C222A_ (Szczesny *et al*., 2010), respectively, using QuikChange (Agilent Technologies Inc., Waldbronn, Germany). WT and mutated coding sequences of *xopB*, *avrBs1*, *xopS* and *xopQ* were introduced into expression vectors by Golden Gate cloning. ‘WT’ and mutated *avrBsT_C222A_* sequences were amplified using pENTR/DavrBsT_C222A_ and pENTR/DavrBsT_C222A;TrM_– as templates, thereby introducing suitable *Bsa*I sites, and cloned into expression vectors by *Bsa*I⁄T4‐ligase cut‐ligation. Similarly, truncated *xopB* and *avrBs1* variants were amplified from pJET:xopB_ns, pJET:avrBs1_ns and respective mutant derivatives and introduced into expression vectors. Cloning details are available on request. The constructs used in this study are listed in Table S2 and the oligonucleotides in Table S3 (see Supporting Information).

### Secretion assay

*Xanthomonas* *in vitro *secretion experiments were performed as described previously (Büttner *et al*., 2002) with the following modifications: bacteria were incubated for 1.5 h in MA medium (pH 5.3); culture supernatants were precipitated with 100% TCA (w/v).

### Translocation assay

Two leaves of three ECW‐30R plants and one ECW‐10R plant (control) were inoculated with bacterial solutions (OD_600_ = 0.04 and OD_600_ = 0.2, respectively) of *Xcv* strains 85*, 85*Δ*hpaB* and 85‐10 ectopically expressing AvrBs3Δ2 (pBR356) derivatives (see Table S2). Plant reactions were scored at 2–4 days post‐inoculation (dpi); scores between 0 and 3 were given based on the strength of visible cell death (Fig. S1). Altogether, four spots per strain and plant, i.e. 12 spots per experiment, were analysed, resulting in a maximum possible score of 36 for a single strain. For better comparison, scores for the strains inducing the strongest reactions, i.e. 85*(pBR356:xopB_1–177_), 85*Δ*hpaB* (pBR356:xopB_1–177;TrM_^–^) and 85‐10 (pBR356:xopB_1–177_), were set to 100% in the respective experiments. Bacterial solutions used for translocation assays were analysed for the expression of AvrBs3Δ2‐FLAG and respective fusion proteins by immunoblot.

### Fractionation studies

Bacteria were grown O/N in 50 mL of MA (pH 7.0) containing appropriate antibiotics. At OD_600_ = 0.8–1.2, cells were harvested by centrifugation (8000 ***g***, 4 °C), resuspended in 50 mm HEPES (pH 7.4) and disrupted by a French pressure cell. Unlysed cells and debris were removed by centrifugation (21 000 ***g***, 5 min, 4 °C). Cell lysates (TE) were adjusted to comparable cell numbers (OD_600_) and separated into soluble (S) and insoluble (M) material by ultracentrifugation (200 000 ***g***, 2 h, 4 °C). Five microlitres of each fraction were analysed by sodium dodecylsulphate‐polyacrylamide gel electrophoresis (SDS‐PAGE) and immunoblot.

### Liposome flotation assay

GST‐tagged proteins were synthesized in *E. coli* BL21 (DE3) RIL cells (2 h induction by 1 mm IPTG, 3% ethanol at room temperature). Cells from 500 mL of culture were resuspended in 20 mL of phosphate‐buffered saline (PBS) with protease inhibitor complete (Sigma‐Aldrich, Munich, Germany) and disrupted via freeze–thaw cycles. After centrifugation (20 000 ***g***, 30 min, 4 °C), soluble GST fusion proteins were immobilized O/N at 8 °C on a glutathione sepharose matrix (2 mL per 500 mL of culture; GE Healthcare, Freiburg, Germany). After three washing steps with approximately 30 mL of PBS each, bound proteins were eluted at 8 °C with three 10‐mL aliquots of elution buffer (100 mm Tris‐Cl, pH 8.5, 50 mm NaCl, 20 mm reduced glutathione). Elution fractions were pooled and concentrated using Amicon Ultra Centrifugal Filter Devices (Sigma‐Aldrich). Glutathione levels were reduced (by at least a factor of 10) by iterative dilution and concentration steps using elution buffer without glutathione. The protein concentration and quality were determined using a NanoDrop ND‐1000 spectrophotometer (Thermo Fisher Scientific, Waltham, MA, USA), and confirmed by SDS‐PAGE and immunoblot.

For liposome preparation, lipid mixtures at different molar ratios were prepared from 1,2‐dioleoyl‐*sn*‐glycero‐3‐phosphocholine (DOPC), 1,2‐dioleoyl‐*sn*‐glycero‐3‐phosphoethanolamine (DOPE), 1,2‐dioleoyl‐*sn*‐glycero‐3‐phospho‐1'‐*rac*‐glycerol (DOPG), sodium salts, and 18 : 1 CL (1′,3′‐bis[1,2‐dioleoyl‐*sn*‐glycero‐3‐phospho]‐*sn*‐glycerol, sodium salt), dissolved in chloroform–methanol 2 : 1 (volume ratio). For lipid quantification, 1 mol.% DiI stain (1,1′‐dioctadecyl‐3,3,3′,3′‐tetramethylindocarbocyanine perchlorate) was added. After evaporation of the solvent, membranes were hydrated by the addition of TN buffer (100 mm Tris, pH 8.5, 50 mm NaCl) to a final lipid concentration of 5 mm. Unilamellar liposomes were extruded by passing the lipid suspension 21 times through a 400‐nm polycarbonate membrane.

For flotation assays, typically, 5 µm GST‐tagged protein was incubated for 1 h at room temperature (RT) with lipid vesicles (1 mm final lipid) in a total volume of 80 µL. For sucrose gradients, samples were mixed with 50 µL of 2.5 m sucrose in TN‐buffer; 110 µL of this mixture were transferred to a centrifuge tube and overlaid first with 70 µL of 0.75 m sucrose in TN‐buffer and, finally, with 20 µL of TN‐buffer. After centrifugation (30 min, 465 000 ***g***, 22 °C), 50 µL of the liposome‐containing upper fraction and 50 µL of the lower fraction were removed. The upper fractions were analysed for lipid content using the DiI ultraviolet/visible (UV/Vis) signal and normalized to contain equal amounts of lipids. The amounts of liposome‐bound and free protein were then analysed by immunoblot.

### Immunoblot analysis

SDS‐PAGE and immunoblotting were performed following standard protocols and primary antibodies as follows. Secretion assays: α‐AvrBs3 (Knoop *et al*., 1991), α‐HrpF (Büttner *et al*., 2002), α‐HrcJ (Rossier *et al*., 2000). Translocation assays: α‐FLAG (mouse; Sigma‐Aldrich). Liposome flotation: α‐GST (goat; GE Healthcare). Fractionation: α‐GFP (mouse; Roche Diagnostics, Mannheim, Germany), α‐XopB (Schulze *et al*., 2012), α‐AcnB (Gruer *et al*., 1997), α‐HrcJ (Rossier *et al*., 2000). Horseradish peroxidase‐labelled secondary antibodies (GE Healthcare) were detected by enhanced chemiluminescence.

### Bioinformatic analysis

N‐terminal sequences (at most 180 amino acids) of all known effectors in *Xcv* 85‐10 and of AvrBs3, AvrBs4, AvrBsT, AvrXv3 and AvrXv4 (for Genbank accession numbers, see Table S1) served as input for a custom motif discovery tool based on profile HMMs (Krogh *et al*., 1994). The architecture of the core HMM was identical to the PLAN7 architecture of HMMer (Eddy, 1998) with 15 match states and additional insert states to represent amino acids preceding and succeeding the core HMM (Fig. S8, see Supporting Information). All insert states were fixed to a uniform distribution, whereas emission probabilities at the match states and transition probabilities were learned from the input sequences by a Bayesian variant of the Baum–Welch algorithm using a difference of 1E‐6 on the log‐posterior value as stop criterion. The motif discovery tool was implemented in Java using the Jstacs library (Grau *et al*., 2012), version 2.3. Motif occurrences were derived from the Viterbi path of each input sequence as those amino acids emitted by the core HMM. Motif occurrences were used as input for a multiple sequence alignment using kalign2 (Lassmann *et al*., 2009), and the resulting alignment was provided to WebLogo (Crooks *et al*., 2004) to produce a sequence logo (Schneider and Stephens, 1990). Motif scores were also derived from the Viterbi path by extracting from each N‐terminal sequence the subsequence emitted by the core HMM, which makes scoring independent of the length of the extracted N‐terminal sequence. The log‐likelihood of each subsequence according to the profile HMM was then divided by its length to account for differences in insert or delete states visited in a Viterbi path, yielding the final relative HMM score.

## Supporting information

Figure S1 Translocation efficiency score

Figure S2 Translocation of Xop_B1‐177_::AvrBs3Δ2 and derivatives by X*cv* strain 85‐10

Figure S3 Plant reactions to transient expression of XopB_1‐177_::AvrBs3Δ2, AvrBs1_1‐91_::AvrBs3Δ2, AvrBsT_C222A_::AvrBs3Δ2 and derivatives

Figure S4 XopB interacts with HpaB *in vitro* independent of the TrM

Figure S5 The TrM is not required for XopB activity in planta

Figure S6 Expression of AvrBs3Δ2 fusion proteins

Figure S7 Protein purification

Figure S8 Structure of the profile HMM for motif discovery. Match states representing the motif are represented by rectangles, delete states by circles, and insert states by diamonds. Insert states “Im” and “In” are responsible for emitting amino acids preceding and succeeding the core motif. Transition probabilities are denoted at the edges

Table S1 TrM occurences in the N‐terminal 180 aa of T3Es and non‐effectors from X*cv*

Table S2 Strains and plasmids used in this study

Table S3 Oligonucleotides used in this study

Supporting Experimental Procedures: Agrobacterium‐mediated expression in planta GST pull‐down assay
