# Supplementary figures and images for: A conserved motif promotes HpaB‐regulated export of type III effectors from Xanthomonas
Source: Mol Plant Pathol. 2018 Oct 16;19(11):2473–87. doi: 10.1111/mpp.12725 (PMC6638074; doi:10.1111/mpp.12725)

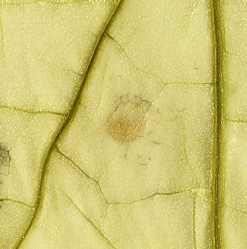

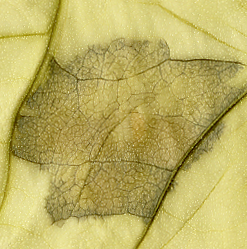

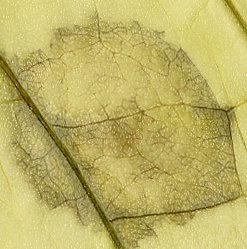

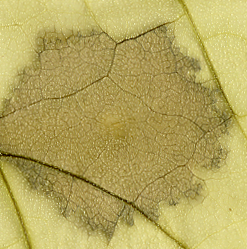

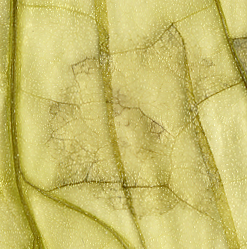

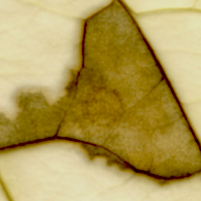

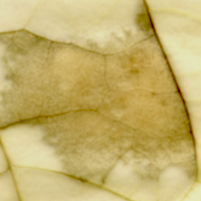

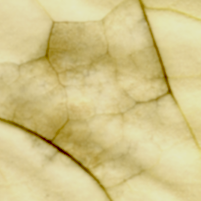

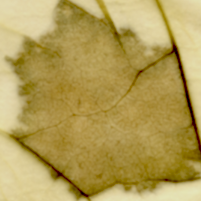

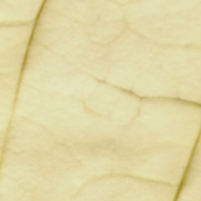


0

0.5

1

2

3

Plant reaction score

leaf 1

leaf 2

ECW-30R

**Figure S1**

Translocation efficiency score.

Supplement: Supplementary file 1 — Figure S1 Translocation efficiency score [file MPP-19-2473-s001.docx]

**
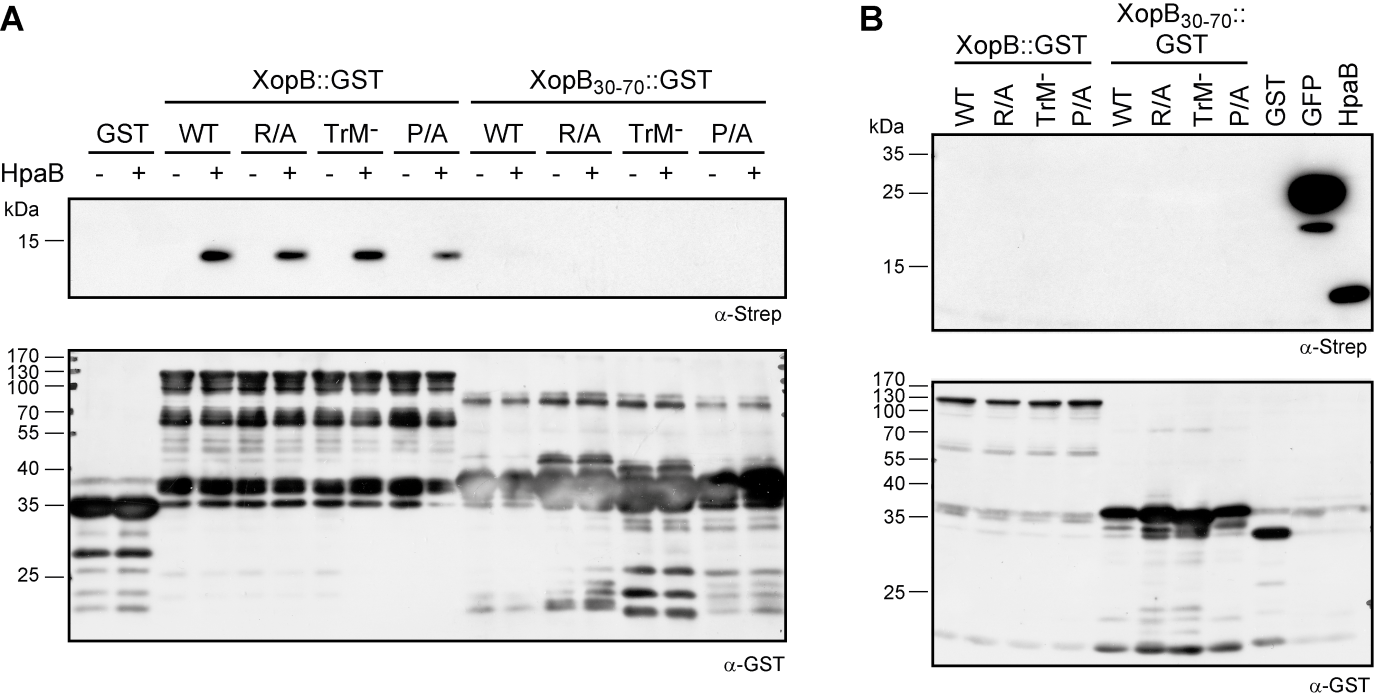
**

**Figure S4**

XopB interacts with HpaB *in vitro* independent of the TrM.

Supplement: Supplementary file 4 — Figure S4 XopB interacts with HpaB in vitro independent of the TrM [file MPP-19-2473-s004.docx]

**
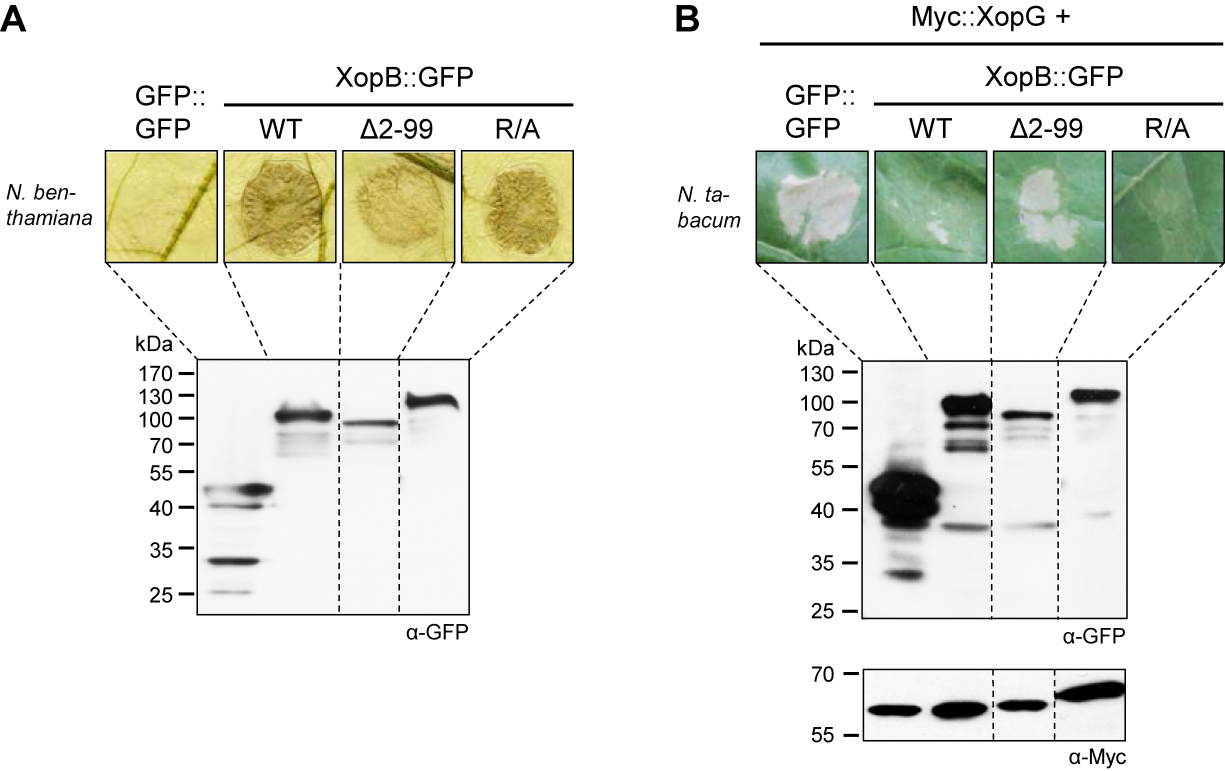
**

**Figure S5**

The TrM is not required for XopB activity *in planta*.

Supplement: Supplementary file 5 — Figure S5 The TrM is not required for XopB activity in planta [file MPP-19-2473-s005.docx]

**
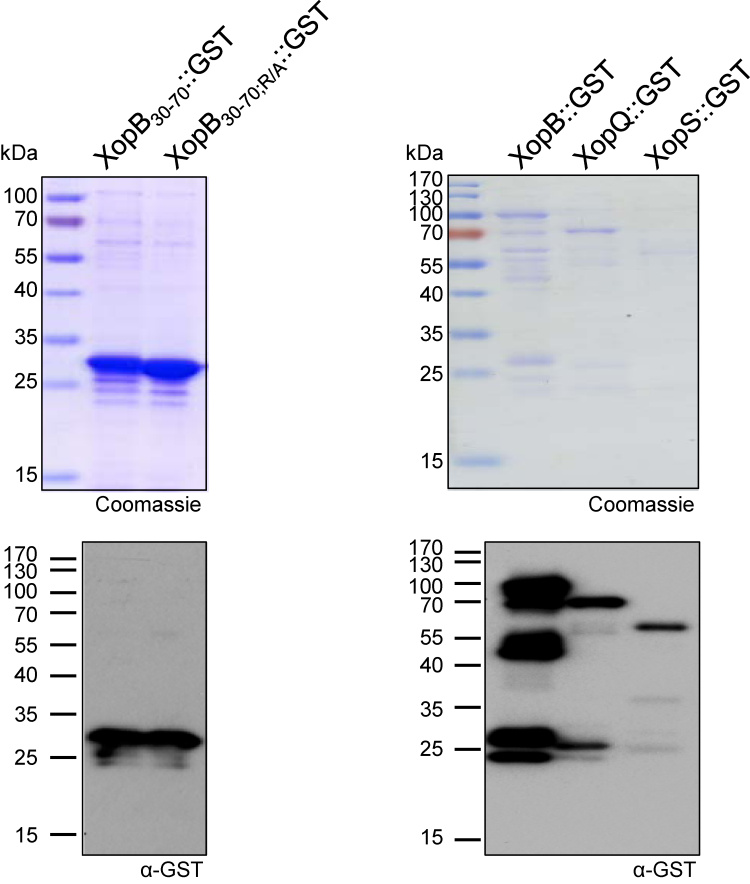
**

**Figure S7**

Protein purification.

Supplement: Supplementary file 7 — Figure S7 Protein purification [file MPP-19-2473-s007.docx]

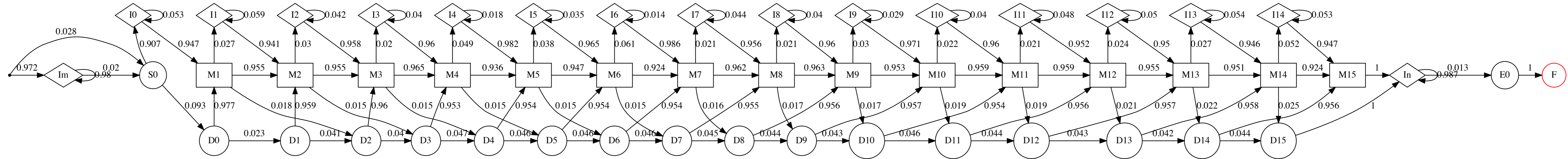

Supplement: Supplementary file 8 — Figure S8 Structure of the profile HMM for motif discovery. Match states representing the motif are represented by rectangles, delete states by circles, and insert states by diamonds. Insert states “Im” and “In” are responsible for emitting amino acids preceding and succeeding the core motif. Transition probabilities are denoted at the edges [file MPP-19-2473-s008.pdf]
