# Supplementary material for: A conserved motif promotes HpaB‐regulated export of type III effectors from Xanthomonas
Source: Mol Plant Pathol. 2018 Oct 16;19(11):2473–87. doi: 10.1111/mpp.12725 (PMC6638074; doi:10.1111/mpp.12725)
Supplement: Supplementary file 2 — Figure S2 Translocation of XopB1‐177::AvrBs3Δ2 and derivatives by Xcv strain 85‐10 [file MPP-19-2473-s002.docx]

**Figure S2**


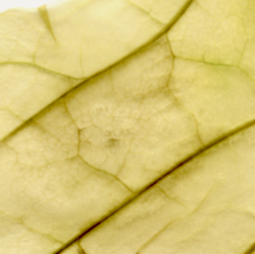

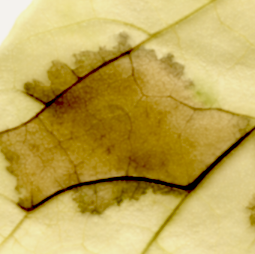

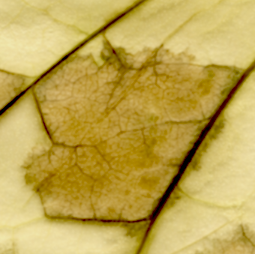

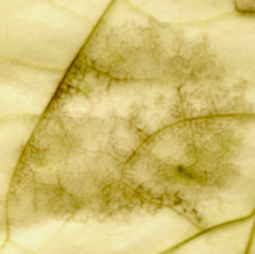

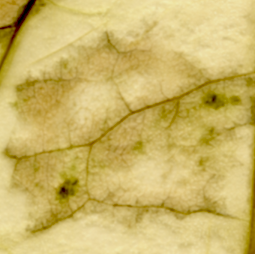

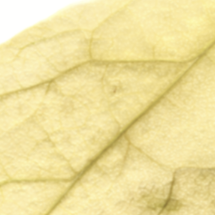

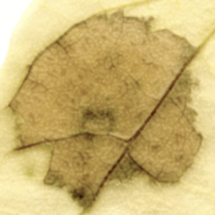

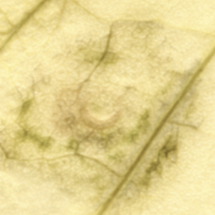

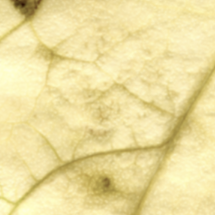

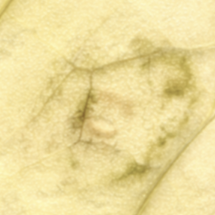

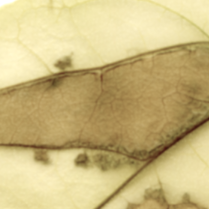

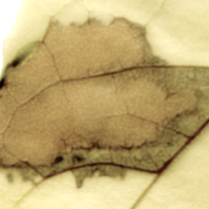

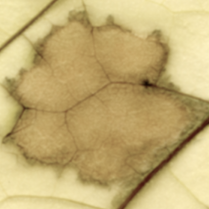

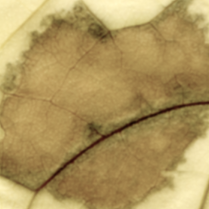

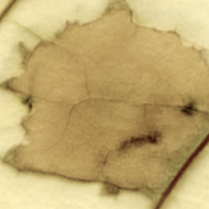

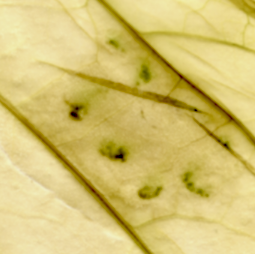

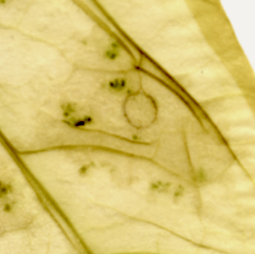

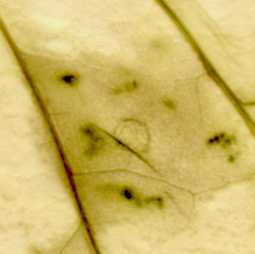

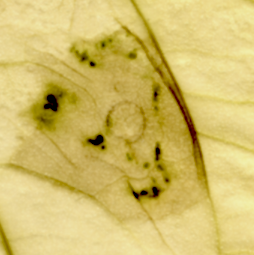

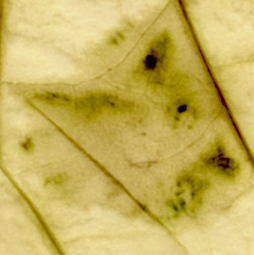


**85-10**

P/A

**A**

0

20

40

60

80

100

120

XopB

TrM^-^

R/A

Relative translocation

efficiency (%)

XopB

TrM^-^

R/A

P/A

-

**B**

**C**

ECW-30R

ECW-10R

ECW-30R

cfu/ml

2.5×10^8^

**85-10**

**85-10Δ*hrpF***

XopB

TrM^-^

R/A

P/A

-

2.5×10^8^

2.5×10^8^

cfu/ml

5×10^7^

Translocation of XopB_1-177_::AvrBs3Δ2 and derivatives by *Xcv* strain 85-10.
