# Supplementary material for: A conserved motif promotes HpaB‐regulated export of type III effectors from Xanthomonas
Source: Mol Plant Pathol. 2018 Oct 16;19(11):2473–87. doi: 10.1111/mpp.12725 (PMC6638074; doi:10.1111/mpp.12725)
Supplement: Supplementary file 3 — Figure S3 Plant reactions to transient expression of XopB1‐177::AvrBs3Δ2, AvrBs11‐91::AvrBs3Δ2, AvrBsTC222A::AvrBs3Δ2 and derivatives [file MPP-19-2473-s003.docx]

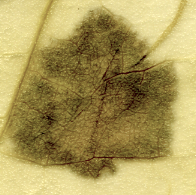

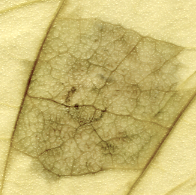

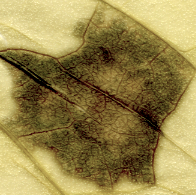

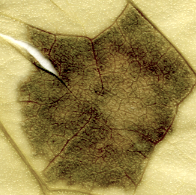

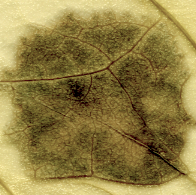

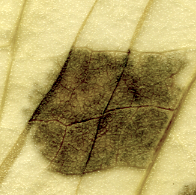

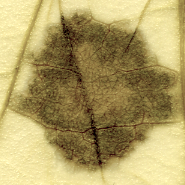

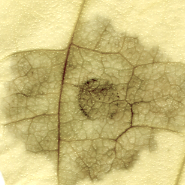

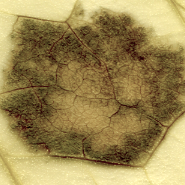

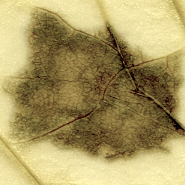

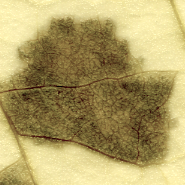

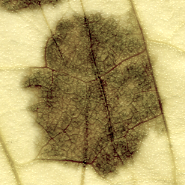


WT

TrM^-^

R/A

P/A

GFP

XopB_1-177_::AvrBs3Δ2

α-AvrBs3

XopB_1-177_

AvrBs3Δ2 fusion

**B**

**A**

**C**

WT

TrM^-^

R/A

P/A

GFP

AvrBs3Δ2

AvrBs3Δ2

GFP

AvrBs3Δ2


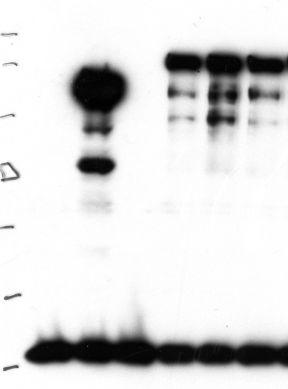

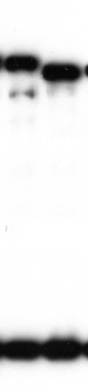

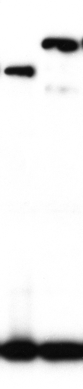

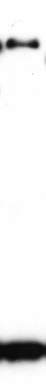


kDa

170

130

100

70

55

40

35

AvrBs1_1-91_

AvrBs1_1-111;TrM_-

AvrBsT_C222A_

AvrBsT_C222A;TrM_-

AvrBs3Δ2 fusion

AvrBs1_1-91_

AvrBs1_1-111;TrM_-

AvrBsT_C222A_

AvrBsT_C222A;TrM_-

**Figure S3**

Plant reactions to transient expression of XopB_1-177_::AvrBs3Δ2, AvrBs1_1-91_::AvrBs3Δ2, AvrBsT_C222A_::AvrBs3Δ2 and derivatives.
