# Supplementary material for: A conserved motif promotes HpaB‐regulated export of type III effectors from Xanthomonas
Source: Mol Plant Pathol. 2018 Oct 16;19(11):2473–87. doi: 10.1111/mpp.12725 (PMC6638074; doi:10.1111/mpp.12725)
Supplement: Supplementary file 6 — Figure S6 Expression of AvrBs3Δ2 fusion proteins [file MPP-19-2473-s006.docx]

**B**

**A**

**C**


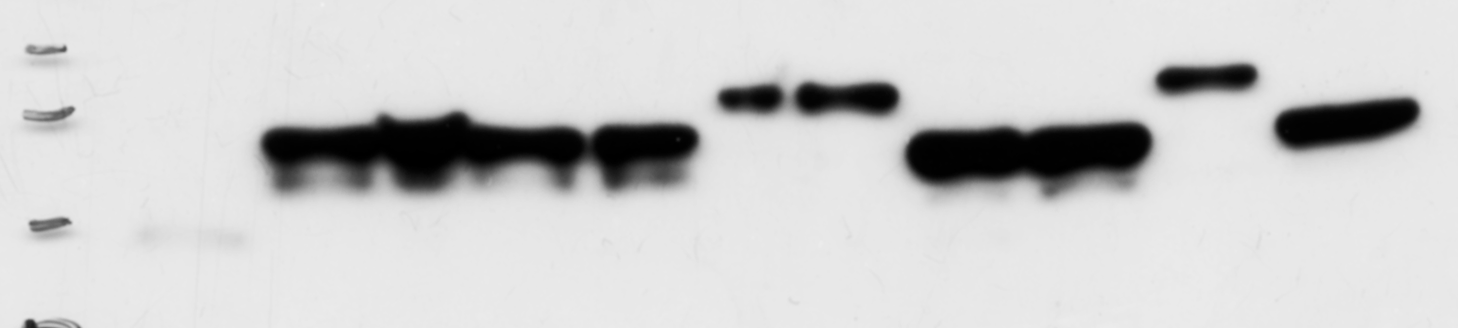


WT

TrM^-^

WT

TrM^-^

α-FLAG

XopB_1-177_

AvrBs3Δ2 fusion

AvrBs1_1-111_

AvrBsT_C222A_

WT

TrM^-^

R/A

P/A

-

kDa

170

130

100

70


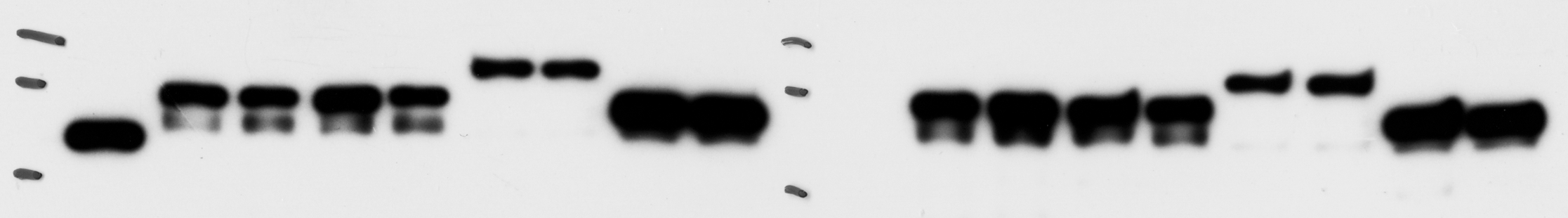


α-FLAG

XopB_1-177_::

AvrBs3Δ2

WT

TrM^-^

R/A

P/A

AvrBs3Δ2

kDa

170

130

100


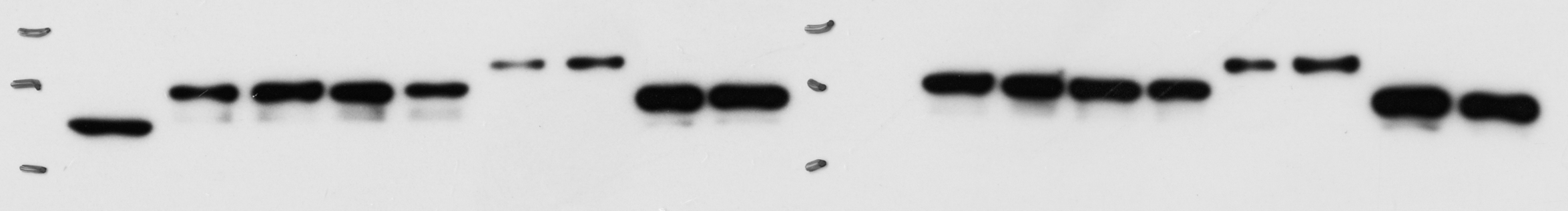


WT

TrM^-^

WT

TrM^-^

α-FLAG

XopB_1-177_

AvrBs3Δ2 fusion

WT

TrM^-^

R/A

P/A

kDa

170

130

100

AvrBs3Δ2


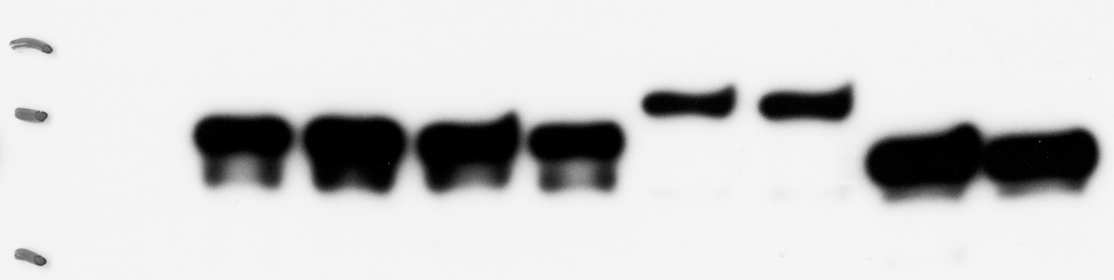


kDa

170

130

100

XopB_1-177_::

AvrBs3Δ2

WT

TrM^-^

R/A

P/A

-

α-FLAG

α-FLAG


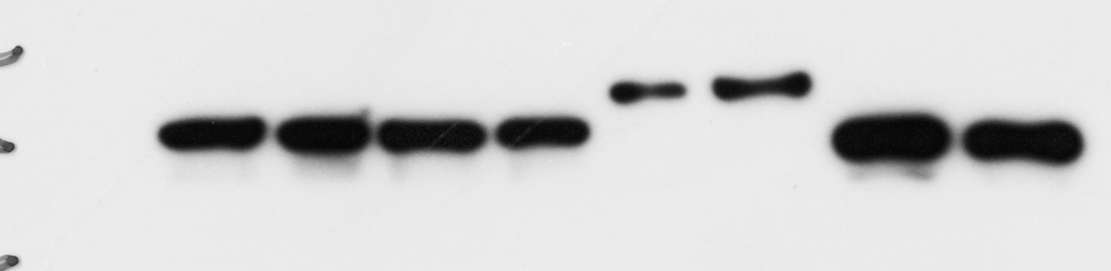


WT

TrM^-^

WT

TrM^-^

XopB_1-177_

AvrBs3Δ2 fusion

WT

TrM^-^

R/A

P/A

kDa

170

130

100

AvrBs3Δ2

**E**

**D**

AvrBs1_1-111_

AvrBsT_C222A_

AvrBs1_1-111_

AvrBsT_C222A_

**Figure S6**

Expression of AvrBs3Δ2 fusion proteins.
