# Supplementary material for: A conserved motif promotes HpaB‐regulated export of type III effectors from Xanthomonas
Source: Mol Plant Pathol. 2018 Oct 16;19(11):2473–87. doi: 10.1111/mpp.12725 (PMC6638074; doi:10.1111/mpp.12725)
Supplement: Supplementary file 9 — Table S1 TrM occurences in the N‐terminal 180 aa of T3Es and non‐effectors from Xcv [file MPP-19-2473-s009.docx]

**Table S1: TrM occurences in the N-terminal 180 aa of T3Es and non-effectors from *Xcv***.

| **Protein^a^** | **Genbank** | **Motif** | | | **HMM score** |
| --- | --- | --- | --- | --- | --- |
|  |  | **Start** | **Stop** | **Sequence** |  |
| **Effectors** | | | | | |
| AvrBs3 | CAA34257 | 37 | 52 | GPLDGLPARRTMSRT | -2.35 |
| AvrBs4 | CAA48680 | 37 | 52 | GPLDGLPARRTMSRT | -2.35 |
| AvrXv4 | AAG39033 | 47 | 62 | SGLASRPRRKAELLA | -2.38 |
| XopV | CAJ22288 | 35 | 50 | SQLRELPRLRRSASA | -2.42 |
| XopP | CAJ22867 | 44 | 59 | GGLSIRPRRLGRASR | -2.43 |
| AvrBs2 | CAJ21683 | 37 | 52 | PPLRERPRRRAGNMP | -2.45 |
| XopAK | CAJ25517 | 89 | 104 | CQLRTSAARRTRSAR | -2.47 |
| XopM | CAJ22073 | 38 | 53 | SPLSALPPRPSGRRN | -2.47 |
| XopX | CAJ22203 | 39 | 55 | PSLAALTPRSRSRASS | -2.47 |
| AvrRxv | CAJ22102 | 69 | 84 | SALPTRPRKKAEALS | -2.48 |
| XopS | CAJ21955 | 64 | 79 | SSLERPARRPSGSRS | -2.5 |
| XopL | CAJ24951 | 133 | 148 | PPVGASPLRRSTALR | -2.52 |
| XopE1 | CAJ21925 | 42 | 57 | PSLQGLARLGAARAR | -2.54 |
| XopN | CAJ24623 | 45 | 60 | SPLGRAPVRRSGANT | -2.54 |
| AvrBsT | AAD39255 | 50 | 65 | SGLPERPRKKAIALE | -2.56 |
| XopAA | CAJ25516 | 85 | 100 | PDLRSQPARSGASQP | -2.58 |
| XopB | CAJ22212 | 46 | 63 | SRLPTTRPPRRRSTSSG | -2.59 |
| XopF1 | CAJ22045 | 39 | 54 | GPLAGLASSSAALRG | -2.6 |
| XopAP | CAJ24869 | 79 | 94 | PGLSAAPVKRGRNAT | -2.61 |
| HpaA | CAJ22051 | 46 | 61 | PRLRPAPPRRRRRGI | -2.62 |
| XopF2 | CAJ24621 | 42 | 58 | KGLAEPSPRLRARAGK | -2.62 |
| AvrBs1 | CAJ19916 | 36 | 51 | PSLDSQALKKAPRKR | -2.63 |
| AvrRxo1 | CAJ26159 | 71 | 86 | TQLDNLPARPASSKS | -2.63 |
| XopK | CAJ24946 | 110 | 125 | KVLQTRPLRRRTRRD | -2.64 |
| XopC | CAJ24112 | 164 | 179 | GRLKTSPLLSTQSQG | -2.65 |
| XopQ | CAJ26169 | 60 | 75 | RRAQSLPARLTPAQR | -2.66 |
| XopAV | CAJ22828 | 140 | 156 | TQLAQSLPRRPTAIST | -2.67 |
| XopAU | CAJ22827 | 109 | 124 | CLLETPARLSALAGA | -2.68 |
| XopO | CAJ22686 | 39 | 54 | PALEGLPSIRQGMQA | -2.69 |
| XopD | DAA34040 | 42 | 57 | PLLESLPRRNPTQVH | -2.7 |
| XopZ | CAJ23736 | 75 | 90 | PGLKAAPKRKHALPR | -2.7 |
| AvrXv3 | AAG18480 | 47 | 63 | VRLASTRIKRSSGAGR | -2.71 |
| XopJ | CAJ23833 | 63 | 78 | SGLPERVALKTKLLA | -2.71 |
| XopE2 | CAJ23957 | 43 | 59 | PSLHGLVALGSSGTRR | -2.72 |
| XopI | CAJ22437 | 135 | 150 | GRLAARIPSLPQAQR | -2.72 |
| XopR | CAJ21916 | 88 | 103 | PALATSAPNAARQGT | -2.72 |
| XopAX | CAJ19898 | 41 | 56 | ELLAEIATRRGTTSA | -2.75 |
| XopG | CAJ22929 | 29 | 44 | SALEKIAAGSSGSAL | -2.75 |
| XopAW | CAJ24824 | 130 | 145 | GELSTLVNGDSTSST | -2.79 |
| XopAD | CAJ26046 | 47 | 62 | SSGAQQPLGRPPRKR | -2.8 |
| XopH | CAJ19917 | 41 | 56 | AELADLPSRQPPRSK | -2.86 |
| **Non-effectors** | | | | | |
| HrpE | CAJ22048 | 22 | 37 | GGLSNGISGAAALSG | -3.05 |
| XopA | CAJ22071 | 100 | 115 | GGLNTSLSLSGDTAS | -3.1 |
| HrpB2 | CAJ22059 | 13 | 28 | SAAATQALSPVATPN | -3.13 |
| HrpF | CAJ22042 | 112 | 127 | KGKDAPPALEGSTVT | -3.17 |

^a^ Order based on HMM score.
