# Supplementary material for: A conserved motif promotes HpaB‐regulated export of type III effectors from Xanthomonas
Source: Mol Plant Pathol. 2018 Oct 16;19(11):2473–87. doi: 10.1111/mpp.12725 (PMC6638074; doi:10.1111/mpp.12725)
Supplement: Supplementary file 10 — Table S2 Strains and plasmids used in this study [file MPP-19-2473-s010.docx]

**Table S2: Strains and plasmids used in this study.**

| **Name** | **Relevant characteristics*** | **Reference/**  **source** |
| --- | --- | --- |
| **Strains** | | |
| ***Xanthomonas campestris*pv.*vesicatoria*** | | |
| 85-10 | Pepper-race 2; wild type; Rp^r^ | (Canteros, 1990, Minsavage *et al.*, 1990) |
| 85-10∆*hpaB* | *hpaB* deletion mutant, derivative of strain 85-10 | (Büttner *et al.*, 2004) |
| 85* | 85-10 derivative containing the *hrpG** mutation | (Wengelnik *et al.*, 1999) |
| 85*∆*hpaB* | *hpaB* deletion mutant, derivative of strain 85* | (Büttner *et al*., 2004) |
| 85*∆*hpaB*∆*hrpF* | *hpaB*/*hrpF* deletion mutant derivative of strain 85* | (Büttner *et al*., 2004) |
| ***Agrobacterium tumefaciens*** | | |
| GV3101 | Carries Ti plasmid pMP90; Rp^r^, Gm^r^ | (Koncz & Schell, 1986) |
| ***Escherichia coli*** | | |
| Top10 | F- *mcr*A Δ(*mrr-hsd*RMS-*mcr*BC) Φ80*lac*ZΔM15 Δ*lac*Χ74 *rec*A1 *ara*D139 Δ(*araleu*) 7697 *gal*U *gal*K *rps*L (Str^R^) *end*A1 *nup*G | Invitrogen, Karlsruhe, Germany |
| BL21 (DE3) RIL | F^−^ *ompT hsdS* (r_B_ ^−^m_B_ ^−^)*dcm* ^+^ Tet^r^ *gal* λ (DE3)*endA* Hte (*argU ileY leuW* Cm^r^) | Agilent Technologies Inc., Santa Clara, U.S.A. |
| **Vectors** | | |
| pBBR1MCS-5 | Broad‐host‐range vector; *lac* promoter; Gm^r^ | (Kovach *et al.*, 1995) |
| pBGWFS7 | Binary expression vector; *att*R1-Cm^r^-*ccdB*-*att*R2-*EGFP::uidA*; Sm^r^ | (Karimi *et al.*, 2005) |
| pBR356 | Broad host range vector containing *avrBs3∆2-FLAG* downstream of the *lac* promoter and the *lacZα* fragment, which is flanked by *Bsa*I sites; Gm^r^ | (Scheibner *et al.*, 2016) |
| pBRM | Golden Gate-compatible broad host range vector; Gm^r^ | (Szczesny *et al.*, 2010b) |
| pGGA1 | Binary expression vector; p*35S* -Cm^r^-*ccdB*-*EGFP*; Sm^r^ | (Schulze *et al.*, 2012) |
| pGGA3 | Golden Gate-compatible derivative of pBGWFS7; binary expression vector; p*35S*-Cm^r^-*ccdB*-FLAG; Sm^r^ | This study |
| pGGA3_356 | Derivative of pGGA3 containing p*35S*-*lacZ*-*avrBs3∆2-FLAG*; Sm^r^ | This study |
| pGGA8 | Binary expression vector; p*35S*-4×*c-myc*-Cm^r^-*ccdB*; Sm^r^ | (Schreiber *et al.*, 2015) |
| pGGE2 | Golden Gate-compatible derivative of pQE-60; *E. coli* expression vector; p*T7*-*GST*-Cm^r^-*ccdB*; Km^r^ | This study |
| pGGE7 | Golden Gate-compatible derivative of pQE-60; *E. coli* expression vector; p*T7-*Cm^r^-*ccdB*-StrepII; Km^r^ | This study |
| pGGE9 | Golden Gate-compatible derivative of pQE-60; *E. coli* expression vector; p*T7*-Cm^r^-*ccdB*-*TEV*-*GST*; Km^r^ | This study |
| pGGX7 | Golden Gate-compatible derivative of  pBBR1MCS-5; broad host range vector; p*lac*-Cm^r^-*ccdB*-*EGFP*; Gm^r^ | This study |
| pGWB2 | Binary expression vector, contains *35S* promoter upstream of *att*R1-Cm^r^-*ccdB*-*att*R2; Hm^r^, Km^r^ | (Nakagawa *et al.*, 2007) |
| pJET2.1blunt | High copy cloning vector; Ap^r^ | Fisher Scientific GmbH, Schwerte, Germany |
| pQE60 | *E. coli* expression vector, Ap^r^ | Qiagen, Hilden, Germany |
| **Constructs for clonings** | | |
| pJET:xopB_ns | Derivative of pJET1.2 containing *xopB* without stop codon flanked by *Bsa*I restriction sites; internal *Bsa*I site in *xopB* removed by SOE-PCR | This study |
| pJET: xopB_Δ2-99__ns | Derivative of pJET1.2 containing *xopB* (codon 100-613) without stop codon flanked by *Bsa*I restriction sites; internal *Bsa*I site in *xopB* removed by SOE-PCR | This study |
| pJET:xopB_R56-58A__ns | Derivative of pJET:xopB_ns bearing mutations generated by SOE-PCR; R56, R57 and R58 of the encoded XopB derivative substituted for A | This study |
| pJET:xopB_P50,54,55A__ns | Derivative of pJET:xopB_ns bearing mutations generated by stepwise site-directed mutagenesis; P50, P54 and P55 of the encoded XopB derivative substituted for A | This study |
| pJET:xopB_TrM_-_ns | Derivative of pJET:xopB_ns bearing mutations generated by stepwise site-directed mutagenesis; aa 50-58 of the encoded XopB derivative substituted for A | This study |
| pJET:avrBs1_ns | Derivative of pJET1.2 containing *avrBs1* without stop codon flanked by *Bsa*I restriction sites | This study |
| pJET:avrBs1_TrM_-_ns | Derivative of pJET:avrBs1_ns bearing mutations generated by stepwise site-directed mutagenesis; P48, R49, K50 and R51 of the encoded AvrBs1 derivative substituted for A | This study |
| pENTR ⁄ DavrBsT_C222A_ | Derivative of pENTR ⁄ D-TOPO containing *avrBsT_C222A_* encoding enzymatically inactive AvrBsT (C222A) | (Szczesny *et al.*, 2010a) |
| pENTR ⁄ DavrBsT_C222A;TrM_- | Derivative of pENTR ⁄ DavrBsT_C222A_ bearing mutations generated by stepwise site-directed mutagenesis; P57, R58, K59 and K60 of the encoded AvrBsT derivative substituted for A | This study |
| pJET:xopQ_ns | Derivative of pJET1.2 containing *xopQ* without stop codon flanked by *Bsa*I restriction sites; internal *Bsa*I site in *xopQ* removed by SOE-PCR | This study |
| pJET:xopS_ns | Derivative of pJET1.2 containing *xopS* without stop codon flanked by *Bsa*I restriction sites | This study |
| **Constructs for expression in *Xcv*** | | |
| pBR356:avrBs1_1-111_ | Derivative of pBR356 encoding AvrBs1_1-111_::AvrBs3∆2-FLAG | This study |
| pBR356:avrBs1_1-111;TrM_- | Derivative of pBR356 encoding AvrBs1_1-111;TrM_-::AvrBs3∆2-FLAG | This study |
| pBR356:avrBsT_C222A_ | Derivative of pBR356 encoding AvrBsT_C222A_::AvrBs3∆2 | This study |
| pBR356:avrBsT_C222A;TrM_- | Derivative of pBR356 encoding AvrBsT_C222A;TrM_-::AvrBs3∆2 | This study |
| pBR356_ev | Derivative of pBR356 containing an in-frame linker encoding KK | This study |
| pBR356:xopB_1-177_ | Derivative of pBR356 encoding XopB_1-177_::AvrBs3∆2-FLAG | (Schulze *et a*l., 2012) |
| pBR356:xopB_1-177;R56-58A_ | Derivative of pBR356 encoding XopB_1-177;R/A_::AvrBs3∆2-FLAG | This study |
| pBR356:xopB_1-177;TrM_^-^ | Derivative of pBR356 encoding XopB_1-177;TrM_-::AvrBs3∆2-FLAG | This study |
| pBR356:xopB_1-177;P50,54,55A_ | Derivative of pBR356 encoding XopB_1-177;P/A_::AvrBs3∆2-FLAG | This study |
| pGGX7:avrBsT_C222A_ | Derivative of pGGX7 encoding AvrBsT_C222A_::GFP | This study |
| pGGX7:avrBsT_C222A;TrM_- | Derivative of pGGX7 encoding AvrBsT_C222A;TrM_-::GFP | This study |
| pGGX7:xopB | Derivative of pGGX7 encoding XopB::GFP | This study |
| pGGX7:xopB_R56-58A_ | Derivative of pGGX7 encoding XopB_R/A_::GFP | This study |
| pGGX7:xopB_TrM_- | Derivative of pGGX7 encoding XopB_TrM_-::GFP | This study |
| pGGX7:xopB_P50,54,55A_ | Derivative of pGGX7 encoding XopB_P/A_::GFP | This study |
| pGGX7:xopQ | Derivative of pGGX7 encoding XopQ::GFP | This study |
| pGGX7:xopS | Derivative of pGGX7 encoding XopS::GFP | This study |
| **Constructs for expression *in planta*** | | |
| pGGA1:GFP | Derivative of pGGA1 encoding GFP::GFP | This study |
| pGGA1:xopB | Derivative of pGGA1 encoding XopB::GFP | This study |
| pGGA1:xopB_Δ2-99_ | Derivative of pGGA1 encoding XopB_Δ2-99_::GFP | This study |
| pGGA1:xopB_R56-58A_ | Derivative of pGGA1 encoding XopB_R/A_::GFP | This study |
| pGGA3:GFP | Derivative of pGGA3 encoding GFP::FLAG | This study |
| pGGA3_356_ev | Derivative of pGGA3_356 containing an in-frame linker encoding KK | This study |
| pGGA3_356:xopB_1-177_ | Derivative of pGGA3_356 encoding XopB_1-177_::AvrBs3Δ2-FLAG | This study |
| pGGA3_356:xopB_1-177;R56-R58A_ | Derivative of pGGA3_356 encoding XopB_1-177;R/A_::AvrBs3Δ2-FLAG | This study |
| pGGA3_356:xopB_1-177;P50,54,55A_ | Derivative of pGGA3_356 encoding XopB_1-177;P/A_::AvrBs3Δ2-FLAG | This study |
| pGGA3_356:xopB_1-177;TrM_- | Derivative of pGGA3_356 encoding XopB_1-177;TrM_-::AvrBs3Δ2-FLAG | This study |
| pGGA3_356:avrBs1_1-91_ | Derivative of pGGA3_356 encoding AvrBs1_1-91_::AvrBs3Δ2-FLAG | This study |
| pGGA3_356:avrBs1_1-111;TrM_- | Derivative of pGGA3_356 encoding AvrBs1_1-111;TrM_-::AvrBs3Δ2-FLAG | This study |
| pGGA3_356:avrBsT_C222A_ | Derivative of pGGA3_356 encoding AvrBsT_C222A_::AvrBs3Δ2-FLAG | This study |
| pGGA3_356:avrBsT_C222A;TrM_- | Derivative of pGGA3_356 encoding AvrBsT_C222A;TrM_-::AvrBs3Δ2-FLAG | This study |
| pGGA8:xopG | Derivative of pGGA8 encoding 4×c-Myc::XopG | This study |
| **Constructs for expression in *E. coli*** | | |
| pGGE2_ev | Derivative of pGGE2 encoding GST | This study |
| pGGE7:GFP | Derivative of pGGE7 encoding GFP::StrepII | This study |
| pGGE7:hpaB | Derivative of pGGE7 encoding HpaB::StrepII | This study |
| pGGE9:xopB | Derivative of pGGE9 encoding XopB::TEV-GST | This study |
| pGGE9:xopB_R56-58A_ | Derivative of pGGE9 encoding XopB_R/A_::TEV-GST | This study |
| pGGE9:xopB_TrM_- | Derivative of pGGE9 encoding XopB_TrM_-::TEV-GST | This study |
| pGGE9:xopB_P50,54,55A_ | Derivative of pGGE9 encoding XopB_P/A_::TEV-GST | This study |
| pGGE9:xopB_30-70_ | Derivative of pGGE9 encoding XopB_30-70_::TEV-GST | This study |
| pGGE9:xopB_30-70;R56-58A_ | Derivative of pGGE9 encoding XopB_30-70;R/A_::TEV-GST | This study |
| pGGE9:xopB_30-70;TrM_- | Derivative of pGGE9 encoding XopB_30-70;TrM_-::TEV-GST | This study |
| pGGE9:xopB_30-70;P50,54,55A_ | Derivative of pGGE9 encoding XopB_30-70;P/A_::TEV-GST | This study |
| pGGE9:xopQ | Derivative of pGGE9 encoding XopQ::TEV-GST | This study |
| pGGE9:xopS | Derivative of pGGE9 encoding XopS::TEV-GST | This study |

* Antibiotic resistances: Ap^r^, ampicillin; Cm^r^, chloramphenicol; Gm^r^, gentamycin; Hm^r^, hygromycin; Km^r^, kanamycin; Rp^r^, rifampicin; Sm^r^, spectinomycin

**References**

**Büttner, D., Gürlebeck, D., Noël, L. and Bonas, U.** (2004) HpaB from *Xanthomonas campestris* pv. *vesicatoria* acts as an exit control protein in type III-dependent protein secretion. *Mol. Microbiol.* **54,** 755-768.

**Canteros, B. J.** (1990) Diversity of plasmids and plasmid-encoded phenotypic traits in *Xanthomonas campestris* pv. *vesicatoria*. *Ph.D. thesis. University of Florida, Gainesville.*

**Karimi, M., De Meyer, B. and Hilson, P.** (2005) Modular cloning and expression of tagged fluorescent protein in plant cells. *Trends Plant Sci.,* **10,** 103-105.

**Koncz, C. and Schell, J.** (1986) The promoter of T_L_-DNA gene *5* controls the tissue-specific expression of chimaeric genes carried by a novel type of *Agrobacterium* binary vector. *Mol. Gen. Genet.,* **204,** 383-396.

**Kovach, M., Elzer, P., Hill, D., Robertson, G., Farris, M., Roop, R.*, et al.*** (1995) Four new derivatives of the broad-host-range cloning vector pBBR1MCS, carrying different antibiotic-resistance cassettes. *Gene,* **166,** 175-176.

**Minsavage, G. V., Dahlbeck, D., Whalen, M. C., Kearney, B., Bonas, U., Staskawicz, B. J.*, et al.*** (1990) Gene-for-gene relationships specifying disease resistance in *Xanthomonas campestris* pv. *vesicatoria*-pepper interactions. *Mol. Plant-Microbe Interact.,* **3,** 41-47.

**Nakagawa, T., Takayuki, K., Hino, T., Tanaka, K., Kawamukai, M., Niwa, Y.*, et al.*** (2007) Development of series of Gateway binary vectors, pGWBs, for realizing efficient construction of fusion genes for plant transformation. *J. Biosci. Bioeng.,* **104,** 34-41.

**Scheibner, F.** (2016) Analyse von Exportsignalen von Typ-III- und Typ-IV-Sekretionssubstraten aus *Xanthomonas campestris* pv. *vesicatoria.* *Ph.D. thesis. Institute for Biology, Martin-Luther University Halle-Wittenberg*.

**Scheibner, F., Schulz, S., Hausner, J., Marillonnet, S. and Büttner, D.** (2016) Type III-dependent translocation of HrpB2 by a nonpathogenic *hpaABC* mutant of the plant-pathogenic bacterium *Xanthomonas campestris* pv. *vesicatoria*. *Appl. Environ. Microbiol.,* **82,** 3331-3347.

**Schreiber, T., Sorgatz, A., List, F., Blüher, D., Thieme, S., Wilmanns, M.*, et al.*** (2015) Refined requirements for protein regions important for activity of the TALE AvrBs3. *PLoS ONE,* **10**, e0120214.

**Schulze, S., Kay, S., Büttner, D., Egler, M., Eschen-Lippold, L., Hause, G.*, et al.*** (2012) Analysis of new type III effectors from *Xanthomonas* uncovers XopB and XopS as suppressors of plant immunity. *New Phytol.,* **195,** 894-911.

**Szczesny, R., Büttner, D., Escolar, L., Schulze, S., Seiferth, A. and Bonas, U.** (2010a) Suppression of the AvrBs1-specific hypersensitive response by the YopJ effector homolog AvrBsT from *Xanthomonas* depends on a SNF1-related kinase. *New Phytol.,* **187,** 1058-1074.

**Szczesny, R., Jordan, M., Schramm, C., Schulz, S., Cogez, V., Bonas, U.*, et al.*** (2010b) Functional characterization of the Xcs and Xps type II secretion systems from the plant pathogenic bacterium *Xanthomonas campestris* pv. *vesicatoria*. *New Phytol.,* **187,** 983-1002.

**Wengelnik, K., Rossier, O. and Bonas, U.** (1999) Mutations in the regulatory gene *hrpG* of *Xanthomonas campestris* pv. vesicatoria result in constitutive expression of all *hrp* genes. *J. Bacteriol.,* **181,** 6828-6831.
