# Supplementary material for: A conserved motif promotes HpaB‐regulated export of type III effectors from Xanthomonas
Source: Mol Plant Pathol. 2018 Oct 16;19(11):2473–87. doi: 10.1111/mpp.12725 (PMC6638074; doi:10.1111/mpp.12725)
Supplement: Supplementary file 11 — Table S3 Oligonucleotides used in this study [file MPP-19-2473-s011.docx]

**Table S3: Oligonucleotides used in this study.**

| **Name** | **Sequence (5´- 3´)** | **Purpose** |
| --- | --- | --- |
| HB_xopB_f2_Bsa | TTTGGTCTCATATGAAGGCAGAGCTCACAC | Generation of pJET:xopB_ns, pBR356:xopB_1-177_, pGGA3_356:xopB_1-177_ and derivatives |
| HB_XopB_r_BsaI | TTTGGTCTCTCACCCGGCTCAGGCGCGGGTTG | Generation of pJET:xopB_ns, pJET:xopB_Δ2-99__ns and pJET:xopB_R56-58A__ns |
| XopB(100-613) fwd/ST | TTTGGTCTCATATGTTGGTGCGCAGGGAGTCGAG | Generation of pJET: xopB_Δ2-99__ns |
| HB_XopB_C444T_f | GGTGTCCCGTCGAGATCCGGAGAAAGCGCTC | SOE-PCR to remove the *Bsa*I site in *xopB* |
| HB_XopB_C444T_r | GAGCGCTTTCTCCGGATCTCGACGGGACACC |  |
| CC_XopB_Mut_PA fwd | GCGGCAGCCAGCACAAGTAG | SOE-PCR to generate pJET:xopB_R56-58A__ns |
| CC_XopB_Mut_PA rev | GGCTGCCGCGGGGGGCCTGG |  |
| HB_xopB_P55A_f | CCAACCACCAGGCCCGCCCGGCGGCGCAGC | Step-wise site-directed mutagenesis to generate pJET:xopB_P50,54,55A__ns |
| HB_xopB_P55A_r | GCTGCGCCGCCGGGCGGGCCTGGTGGTTGG |  |
| HP_xopB_P54+P55A_f | GCCAACCACCAGGGCCGCCCGGCGGCGCAG |  |
| HP_xopB_P54+P55A_r | CTGCGCCGCCGGGCGGCCCTGGTGGTTGGC |  |
| HP_xopB_P50+P54+P55A_f | GAAGATAGCAGATTGGCAACCACCAGGGC |  |
| HP_xopB_P50+P54+P55A_r | GCCCTGGTGGTTGCCAATCTGCTATCTTC |  |
| HB_xopB_P55-R58A_f | CCAACCACCAGGCCCGCCGCGGCAGCCAGC | Step-wise site-directed mutagenesis to generate pJET:xopB_TrM_-_ns |
| HB_xopB_P55-R58A_r | GCTGGCTGCCGCGGCGGGCCTGGTGGTTGG |  |
| HB_xopB_P50A_f | GAAGATAGCAGATTGGCAACCACCAGGCC |  |
| HB_xopB_P50A_r | GAAGATAGCAGATTGGCAACCACCAGGCC |  |
| HB_xopB_T51-P54A_f | GAAGATAGCAGATTGGCAGCCGCCGCGGCCGCCGCGGCAGCCAGCACAAG |  |
| HB_xopB_T51-P54A_r | CTTGTGCTGGCTGCCGCGGCGGCCGCGGCGGCTGCCAATCTGCTATCTTC |  |
| CC_XopB 177_Bsa rev | TTTGGTCTCTGATCTGTCCGCTCTGAGGAGTTG | Generation of pBR356:xopB_1-177_, pGGA3_356:xopB_1-177_ and derivatives |
| CC XopB 30-70 fwd | TTTGGTCTCATATGCTAGAAGTTGGCGCACAGCC | Generation of pGGE9:xopB_30-70_ and derivatives |
| CC XopB 30-70 rev | TTTGGTCTCTCACCAGTGGACTTGGGGCTAGTAGC |  |
| pj_avrbs1_fw | TTTGGTCTCTTATGTCCGACATGAAAGTTAATTTCTC | Generation of pJET:avrBs1_ns |
| pj_avrbs1_ns_rev | TTTGGTCTCTCACCCGCTTCTCCTGCATTTGTAACATG |  |
| HP_avrBs1_111_GATC_r | TTTGGTCTCTGATCATTTCTTTTTATTAGCAATTCCTTG | Generation of pBR356:avrBs1_1-111_ and pBR356:avrBs1_1-111;TrM_- and pGGA3_356:avrBs1_1-111;TrM_- |
| HP_avrBs1_91_GATC_r | TTTGGTCTCTGATCTCTACCATTTATCTTAGCCAAC | Generation of pGGA3_356:avrBs1_1-91_ |
| HB_avrBs1_P48A+R49A_f | CACAAGCCTTGAAAAAAGCAGCTGCAAAAAGAGTAATAAAAGG | Step-wise site-directed mutagenesis to generate pJET:avrBs1_TrM_-_ns |
| HB_avrBs1_P48A+R49A_r | CCTTTTATTACTCTTTTTGCAGCTGCTTTTTTCAAGGCTTGTG |  |
| HP_avrBs1_K50A+R51A_f | CAAGCCTTGAAAAAAGCAGCTGCAGCAGCAGTAATAAAAGAAAATATAGCTGC |  |
| HP_avrBs1_K50A+R51A_r | GCAGCTATATTTTCTTTTATTACTGCTGCTGCAGCTGCTTTTTTCAAGGCTTG |  |
| HB_avrBsT_TATG_f | TTTGGTCTCATATGAAGAATTTTATGCGTTC | Generation of pBR356: avrBsT_C222A_, pGGA3_356: avrBsT_C222A_ and derivatives |
| HB_avrBsT_GATC_r | TTTGGTCTCTGATCTGATTCAATAGTTTTCCTAATTTTC |  |
| HB_avrBsT_P57A+R58A_f | CTCCCGGAACGCGCAGCCAAAAAAGCTATTGCGC | Step-wise site-directed mutagenesis to generate pENTR ⁄ DavrBsT_C222A;TrM_- |
| HB_avrBsT_P57A+R58A_r | GCGCAATAGCTTTTTTGGCTGCGCGTTCCGGGAG |  |
| HP_avrBsT_K59+K60A_f | GGAACGCGCAGCCGCAGCAGCTATTGCGCTGGAAGAATCG |  |
| HP_avrBsT_K59+K60A_r | CGATTCTTCCAGCGCAATAGCTGCTGCGGCTGCGCGTTCC |  |
| xopQ_fw | TTTGGTCTCTTATGCAGCCCACCGCAATCCGTTC | Generation of pJET:xopQ_ns |
| xopQ_ns_rev | TTTGGTCTCTCACCGCGCCCGCGTTGCCCCTCGTCCTG |  |
| xopQ_int1_fw | CAGGGACGCCGATGGACTCGTGCAAC | SOE-PCR to remove the *Bsa*I site in *xopQ* |
| xopQ_int1_rev | GTTGCACGAGTCCATCGGCGTCCCTG |  |
| OM_xopS_fwd | TTTGGTCTCTTATGGGAGATGTACAAATGGGA | Generation of pJET:xopS_ns |
| OM_xopS_rev | TTTGGTCTCTCACCAGAAATGCCATCGCTGGC |  |
| HB_xopG_f_BsaI | TTTGGTCTCATATGCCAATCAGTCAAACAAAC | Generation of pGGA8:xopG |
| HB_xopG_rS_BsaI | TTTGGTCTCTAAGCTTCACATGCCGTGAGGC |  |
| HP_hpaB_f_BsaI | TTTGGTCTCATATGATGAGCAGCGCGCGATTCG | Generation of pGGE7:hpaB |
| HP_hpaB_r_-s_BsaI | TTTGGTCTCTCACCGGCGCGTAACCACAGATAG |  |
| linker-156M-for | TTTGGTCTCTTATGAAAAAAGATCAGAGACCAAA | KK-linker module for “empty” vectors (Scheibner, 2016) |
| linker-156M-rev | TTTGGTCTCTGATCTTTTTTCATAAGAGACCAAA |  |
| OM_EV_F/SH | TATGTGATAATAG | Generation of pGGE2_ev |
| OMS_EV_R/SH | AAGCTATTATCA |  |
