# Supplementary material for: A conserved motif promotes HpaB‐regulated export of type III effectors from Xanthomonas
Source: Mol Plant Pathol. 2018 Oct 16;19(11):2473–87. doi: 10.1111/mpp.12725 (PMC6638074; doi:10.1111/mpp.12725)
Supplement: Supplementary file 12 — Supporting Experimental Procedures: Agrobacterium‐mediated expression in planta GST pull‐down assay [file MPP-19-2473-s012.docx]

**Supporting Experimental Procedures**

***Agrobacterium*-mediated expression *in planta***

*N. benthamiana* and *N. tabacum* plants were inoculated with *Agrobacterium* as described in Experimental Procedures. 2 dpi, two leaf discs per sample were harvested and ground in liquid nitrogen. 150 µl 2×Laemmli buffer were added and 15 µl were analyzed by SDS-PAGE and immunoblotting using α-AvrBs3 (Knoop et al., 1991), α-GFP (rabbit; ThermoScientific) and α-Myc (mouse; Roche Diagnostics, Mannheim, Germany) antibodies.

**GST pull-down assay**

GST- and Strep-tagged proteins were synthesized in BL21 (DE3) RIL cells [2 h induction by 1 mM IPTG, 3% EtOH at room temperature (RT)]. Bacterial cells from 50 ml cultures were resuspended in 1.8 ml PBST with protease inhibitor Complete (Roche Diagnostics) and disrupted via freeze-thaw cycles. Insoluble material was removed by centrifugation (20,000 g, 30 min, 4 °C); GST-fusion proteins from 800 µl soluble fraction (load) were immobilized on 25 µl glutathione sepharose matrix (GE Healthcare, Freiburg, Germany) O/N at 8°C. After two washing steps with 1 ml PBST each, 600 µl soluble fraction containing Strep-tagged interactor was added and incubated for 2 h at 8°C. After washing twice with 1 ml PBST, bound proteins were eluted with 40 µl elution buffer (100 mM Tris-Cl pH 8.5, 50 mM NaCl, 20 mM reduced glutathione) at RT for 1.5 h. 5 µl load and 15 µl elution fraction were analyzed by SDS-PAGE and immunoblotting using *Strep*-Tactin® horse radish peroxidase conjugate (IBA GmbH, Göttingen, Germany) and α-GST (goat; GE Healthcare) antibodies.
